# Efficient clofilium tosylate-mediated rescue of POLG-related disease phenotypes in zebrafish

**DOI:** 10.1038/s41419-020-03359-z

**Published:** 2021-01-19

**Authors:** Nicola Facchinello, Claudio Laquatra, Lisa Locatello, Giorgia Beffagna, Raquel Brañas Casas, Chiara Fornetto, Alberto Dinarello, Laura Martorano, Andrea Vettori, Giovanni Risato, Rudy Celeghin, Giacomo Meneghetti, Massimo Mattia Santoro, Agnes Delahodde, Francesco Vanzi, Andrea Rasola, Luisa Dalla Valle, Maria Berica Rasotto, Tiziana Lodi, Enrico Baruffini, Francesco Argenton, Natascia Tiso

**Affiliations:** 1 Department of Biology, University of Padova, Padova, 35131 Italy; 2 Department of Biomedical Sciences, University of Padova, Padova, 35131 Italy; 3 Department of Cardio-Thoraco-Vascular Sciences and Public Health, University of Padova, Padova, 35128 Italy; 4 Faculty of Biology, University of Barcelona, Barcelona, 08028 Spain; 5 Department of Biology and LENS, University of Florence, Sesto Fiorentino (FI), 50019 Italy; 6 Department of Biotechnology, University of Verona, Verona, 37134 Italy; 7 Institut de Biologie Intégrative de la Cellule (I2BC), Gif-sur-Yvette, 91190 France; 8 Department of Chemistry, Life Sciences and Environmental Sustainability, University of Parma, Parma, 43124 Italy

**Keywords:** Energy metabolism, Disease model, Experimental models of disease

## Abstract

The DNA polymerase gamma (Polg) is a nuclear-encoded enzyme involved in DNA replication in animal mitochondria. In humans, mutations in the *POLG* gene underlie a set of mitochondrial diseases characterized by mitochondrial DNA (mtDNA) depletion or deletion and multiorgan defects, named POLG disorders, for which an effective therapy is still needed. By applying antisense strategies, ENU- and CRISPR/Cas9-based mutagenesis, we have generated embryonic, larval-lethal and adult-viable zebrafish Polg models. Morphological and functional characterizations detected a set of phenotypes remarkably associated to POLG disorders, including cardiac, skeletal muscle, hepatic and gonadal defects, as well as mitochondrial dysfunctions and, notably, a perturbed mitochondria-to-nucleus retrograde signaling (CREB and Hypoxia pathways). Next, taking advantage of preliminary evidence on the candidate molecule Clofilium tosylate (CLO), we tested CLO toxicity and then its efficacy in our zebrafish lines. Interestingly, at well tolerated doses, the CLO drug could successfully rescue mtDNA and Complex I respiratory activity to normal levels, even in mutant phenotypes worsened by treatment with Ethidium Bromide. In addition, the CLO drug could efficiently restore cardio-skeletal parameters and mitochondrial mass back to normal values. Altogether, these evidences point to zebrafish as a valuable vertebrate organism to faithfully phenocopy multiple defects detected in POLG patients. Moreover, this model represents an excellent platform to screen, at the whole-animal level, candidate molecules with therapeutic effects in POLG disorders.

## Introduction

In vertebrates, from fish to humans, the mitochondrial DNA (mtDNA) is replicated by the DNA polymerase gamma (Polg), encoded by the nuclear genome. Mutations in the human *POLG* gene cause a series of mitochondrial diseases with Mendelian inheritance, collectively named POLG-related disorders, characterized by mtDNA depletion (MDD) or accumulation of multiple deletions. These disorders mainly include Alpers–Huttenlocher syndrome (OMIM 203700), mitochondrial neurogastrointestinal encephalopathy syndrome (MNGIE type) (OMIM 613662), mitochondrial recessive ataxia syndrome (SANDO, SCAE) (OMIM 607459), autosomal recessive progressive external ophthalmoplegia (arPEO) (OMIM 258450) and autosomal dominant progressive external ophthalmoplegia (adPEO) (OMIM 157640). To date, about 300 pathogenic mutations have been reported in the Human DNA Polymerase Gamma Mutation Database (https://tools.niehs.nih.gov//polg/). The mutations are associated with a spectrum of clinical presentations, ranging from infantile-onset epilepsies, liver failure, polyneuropathy, ataxia, dilated/hypertrophic cardiomyopathy, male infertility and premature menopause, to late-onset ophthalmoplegia and muscle weakness. To a limited extent, clinical phenotypes correlate with the mtDNA genotype.

It must be considered that approximately 1% of the population is carrier of a *POLG* mutation, indicating that the potential frequency of POLG-related disorders is about 1/10,000; in fact, it has been estimated that 10% of adult mitochondrial diseases and 10–25% of PEO are caused by mutations in *POLG*, and that the present underestimation of the reported cases will be corrected due to amelioration and precocity of the diagnosis^1^.

Ample information linked to *POLG* mutations has been obtained from studies on the orthologous gene *MIP1* in the yeast *Saccharomyces cerevisiae*. This species is a very suitable model organism for the study of mitochondrial diseases, since it can survive and grow with MDD or large deletions. Mutant cells produce small colonies, called “*petite*”; the frequency of *petite* mutant onset is a direct index of mtDNA instability. Moreover, the yeast has been a fundamental organism for providing the first evidence that loss of mtDNA can trigger distinct changes in nuclear gene expression, in a molecular cross-talk known as “mitochondria-to-nucleus retrograde signalling”^2^. Since then, several mitochondria-to-nucleus communications have been identified and characterized, among which Hif (Hypoxia-Inducible Factor) and CREB (cAMP response element-binding protein) signalling pathways, both highly relevant in physiological metabolism and human disease^3^. For all these reasons, the yeast plays an important role as a model organism for the study of mitochondrial dysfunction and the validation of pathological mutations in *POLG*^4^.

As far as concerns the animal models for POLG disease, the roundworm *Caenorhabditis elegans*, mutated in the corresponding *polg-1* gene, has proved to be a good system to study Polg deficiency and MDD^5^. Conversely, mouse Polg mutants produced so far loose viability in embryogenesis; a knockin transgenic line shows premature aging (“mutator mouse”), while mouse lines with heart- or brain-specific mutations do not fully recapitulate the entire spectrum of human disease phenotypes^1^.

In the last years, a zebrafish (*Danio rerio*) *polg* mutant has been described by Rahn et al.^6^, developing severe MDD, but dying at juvenile stage (4 weeks post-fertilization), thus hindering further observations at adult stages. A neutrophil-specific zebrafish *polg* knockout is also available^7^, limiting, however, the analysis of *polg* function only in this cell type.

To date, there are no evidence-based therapies for POLG-related disorders, and no randomized controlled clinical trials have been performed. The main treatment is based on symptomatic therapy and supportive care^1^. Thus, the identification of drugs, effective in the rescue of MDD, is urgently needed.

While performing a large-scale drug screen in yeast and worm Polg models, members of our collaboration group succeeded in identifying a compound, Clofilium tosylate (CLO), able to rescue the mutant phenotypes and, when tested in fibroblasts from human POLG patients, effective in increasing mtDNA levels^8^. As shown in the present study, these preliminary results prompted us to test the CLO efficacy at the whole vertebrate animal level, upon generation of zebrafish-based models for POLG disorders. In particular, the zebrafish line *polg*^*sa9574*^, bearing a hypomorphic nonlethal point mutation in the *polg* gene, could allow the long-term analysis of Polg-related phenotypes and the evaluation of CLO therapeutic effects at both larval and adult stages.

## Material and methods

### Maintenance and handling of zebrafish lines

All experiments were performed in accordance with the Italian and European Legislations (Directive 2010/63/EU)^9^ and with permission for animal experimentation from the Ethics Committee of the University of Padua and the Italian Ministry of Health (Authorization number 407/2015-PR). *Danio rerio* (zebrafish) were maintained in a temperature-controlled (28.5 °C) environment in a 12:12 light-dark (LD) cycle and fed as described by Kimmel and colleagues^9,10^. For anaesthesia or euthanasia of zebrafish embryos and larvae, tricaine (MS222; E10521, Sigma–Aldrich) was added to the fish water at 0.16 mg/mL or 0.3 mg/mL, respectively. Wild-type (wt) lines used in this work included Tuebingen, Giotto and Umbria strains^11^. For in vivo studies, the following transgenic lines were used: liver-expressed *Tg(lfabf:dsRed;elaA:EGFP)*^*gz15* 12^; mitochondria-expressed *Tg(Hsa.Cox8a:MLS-EGFP)*^*ia301* 13^; myocardium-expressed *Tg(tg:EGFP,myl7:EGFP)*^*ia300* 14^; CREB signalling reporter *Tg(6xCRE:EGFP)*^14^; Hif-Hypoxia signalling reporter *Tg(4xHRE-TATA:EGFP)*^*ia21* ^^15^.

### Morpholino-mediated *polg* knockdown

For *polg* gene knockdown, antisense morpholino (MO) oligomers were designed and synthesized by GeneTools, targeting the ATG region and the exon4–intron4 boundary of the immature transcript (Supplementary Table 1). MOs were diluted to 2 μg/μL in 1x Danieau buffer (58 mM NaCl, 0.7 mM KCl, 0.4 mM MgSO_4_, 0.6 mM Ca(NO_3_)_2_, 5 mM Hepes, pH 7.6) plus 1% phenol red. For microinjection experiments, 1-cell stage embryos were microinjected with 5 nL of solution. MO-injected embryos (morphants) were raised in fish water with 0.003% PTU (P7629, Sigma) to prevent pigmentation, and analysed within 3 days post-fertilization (dpf). Analysis of MO effects on targeted transcripts (decrease and/or size alteration) was performed by quantitative RT-PCR from RNA of 2 and 3 dpf morphants (primers listed in Supplementary Table 1), followed by agarose gel electrophoresis and signal quantification using the “Measurements” tool of the Volocity 6.0 software (Perkin Elmer).

### Whole-mount in situ hybridization (WISH)

Analysis of embryonic cardiac chambers was performed by WISH using standard protocols^16^ and the following probes: *myh6* (myosin, heavy chain 6, cardiac muscle, alpha) (ZDB-GENE-031112-1) for the atrium, and *myh7* (myosin heavy chain 7) (ZDB-GENE-991123-5) for the ventricle.

### Generation and genotyping of zebrafish *polg* mutant lines

The zebrafish mutant line *polg*^*ia302*^, bearing a 16-nucleotide deletion in the *polg* gene, was generated by CRISPR/Cas9-mediated genome editing. A single guide RNA (sgRNA) was designed using the CHOPCHOP software (https://chopchop.rc.fas.harvard.edu), to specifically target an optimal CRISPR sequence on exon 2 of *polg* gene (XM_001921095). The chosen sgRNA (see Supplementary. Table 1) was generated according to Gagnon et al.^17^ and transcribed in vitro using the MEGAshortscript T7 kit (AM1354, Life Technologies). One-cell stage embryos were injected with 2 nL of a solution containing 280 ng/μL of Cas9 protein (M0646, New England Biolabs) and 68 ng/μL of sgRNA; phenol red was used as injection marker. F0 injected embryos were raised to adulthood and screened, by F1 genotyping, for germline transmission of the mutation. Heterozygous mutants, harbouring the mutation of choice, were outcrossed 4 times and then incrossed to obtain homozygous mutants (F5 generation). Screening primers for heterozygous and homozygous fish (Supplementary Table 1), were designed to amplify a 151-bp region across the *polg* sgRNA target region. PCR products were resolved with ethidium bromide-stained 3% agarose low EEO gel (BP160-500, Fisher BioReagents) to identify *polg*^*+/+*^, *polg*^*+/ia302*^ and *polg*^*ia302/ia302*^ samples.

*Polg*^*sa9574/sa9574*^ point mutants (fish line ZDB-ALT-130411-5185) were identified by HRMA or sequencing of a 108-bp fragment which includes the *polg*^*sa9574*^ point mutation, obtained by genomic DNA PCR amplification (primers in Supplementary Table 1). Genomic DNA was extracted using the HotSHOT protocol^17^ from single larvae, euthanized with a lethal dose of tricaine, or from fin clips. In the latter case, adult fish were anesthetized with tricaine, and biopsies from the caudal fin were removed using a sharp blade.

### Birefringence assay

Muscle birefringence, linked to myofibril organization, was analysed by placing anesthetized embryos, mounted in 2% methyl cellulose, on a glass polarizing filter and covering with a second polarizing filter in a Leica M165FC microscope. Embryos were photographed in bright field with a DFC7000T digital camera. The top filter was twisted until it was possible to see the light refracting through the striated muscle. Pixel intensity in the trunk region was measured with ImageJ software. Values were normalized for samples area because of the slight differences in the size between mutants and wt in analysed stages. Three independent clutches of 10 larvae per genotype and treatment were analysed for their muscle birefringence.

### Survival analysis

We crossed *polg*^*sa9574/sa9574*^ with *polg*^*sa9574/sa9574*^ and wt with wt, respectively, so we had pools of homozygous mutants and wt controls that we raised in different tanks under the same conditions (water, light, food). Survival analysis was carried out by counting the alive individuals once per week, from 3 dpf until 3 months of age.

### Histological analysis

*polg*^*sa9574/sa9574*^ and wt zebrafish were fixed for 24 h in Bouin’s solution at room temperature. The samples were dehydrated through graded series of ethanol, infiltrated with xylene and embedded in Paraplast plus (39602004, Leica). The samples were serially cut into 7–8-μm sections on a LKB microtome. After rehydration, the sections were stained with haematoxylin and eosin and mounted with Eukitt (09-00100, Bio Optica) for microscopic examination.

### Liver analysis

We measured the volume and the integrated density (IntDen) of the fluorescent livers belonging to liver-specific transgenic larvae *Tg(lfabf:dsRed;elaA:EGFP)*^*gz15*^ at 8 and 16 dpf. Measures were done using ImageJ processing imaging software, and data were analysed and represented by GraphPad.

### Transmission electron microscopy (TEM) analysis

Larvae were anaesthetized and fixed overnight in 2% PFA, 2.5% glutaraldehyde in 0.1 M Na cacodylate pH 7.4, rinsed in 0.1 M Na cacodylate buffer, and then included in Epo812 resin. Subsequent TEM steps were performed as described in Giuliodori et al.^14^. Images were acquired with Philips TEM CM 12.

### Mitochondrial DNA quantification

DNA samples, extracted from 10 pooled larvae at 3, 6 and 10 dpf, were prepared as described by Rahn et al.^6^ and quantified by PicoGreen assay (Invitrogen) using the Infinite 200 PRO plate reader (Tecan). Then, 8.5 ng of each sample were used in a qPCR reaction and run in triplicate, using 5× HOT FIREPol® EvaGreen® qPCR Supermix (Solis Biodyne). Mitochondrial DNA copy number was determined by the RT-qPCR standard curve method using *mt-nd1* as the mitochondrial gene target and the *polg* gene as a reference for nuclear DNA, following the protocol developed by Artuso and colleagues^18^.

### RNA extraction and RT-qPCR

Total mRNA was extracted from 30 larvae using Trizol (Invitrogen) and reverse-transcribed using SuperScript® III First-Strand (Thermo Fisher Scientific). Quantitative PCR (qPCR) was performed in triplicated using a Rotor-gene Q (Qiagen) and 5× HOT FIREPol® EvaGreen® qPCR Mix Plus (Solis BioDyne), following the manufacturers’ protocols. The cycling parameters were 95 °C for 14 min, followed by 45 cycles at 95 °C for 15 s, 59 °C for 20 s and 72 °C for 20 s. Threshold cycles (Ct) and dissociation curves were generated automatically by Rotor-Gene Q series software. Sample Ct values were normalized with Ct values from zebrafish *actb2* and *rplp0*, used as control genes. Primer sequences are listed in Supplementary Table 1. All primers were designed using the software Primer3^19^.

### Locomotion assay

Behavioural experiments were performed using the DanioVision tracking system (Noldus Information Technology, Wageningen, The Netherlands). Larvae at 6 and 15 dpf were placed in 48-well plates, with one larva per well in 500 μL of fish water. After 20 min of acclimation, movements of larvae were recorded repeating three cycles of 10 min of light and 10 min of dark, as previously described in MacPhail et al.^20^.

### Ejaculate collection

Fish were isolated for 2 days before stripping, to guarantee the replenishment of sperm reserve. Males were anesthetized in Tricaine 1X (0.16 mg/mL), gently dried on a paper towel and placed in a dampened sponge ventral side up, with their genital papilla exposed. The ventral surface of the fish was further dried to remove any excess water that could prematurely activate the sperm. The fish abdomen was gently pushed with soft plastic tweezers and the ejaculate was collected from the genital papilla by means of a Drummond microdispenser equipped with a 5 μL microcapillary tube. The whole ejaculate was diluted and preserved until analyses (within 1 hour) in 20 μL of zebrafish sperm immobilizing solution (ZSI: 140 mM NaCl, 10 mM KCl, 2 mM CaCl_2_, 20 mM HEPES, pH=8.5)^21^. A total number of 21 males, 8 belonging to *polg*^*+/+*^ and 13 to *polg*^*sa9574/sa9574*^ were stripped. Three males belonging to the *polg*^*sa9574/sa9574*^ line did not produce any ejaculate and, thus, a value of zero was attributed to these males when analysing sperm concentration.

### Sperm concentration

Sperm counts were performed using an improved Neubauer haemocytometer under ×400 magnification, after properly diluting 2 μL of a subsample taken from the whole ejaculate maintained in ZSI. The sample was gently mixed with a micropipette before filling the chamber. The average of five counts per sample was used to estimate sperm concentration, considering dilution steps, and expressed as number of spermatozoa/mL.

### Sperm viability

Sperm viability was measured as the proportion of alive sperm using a Live/Dead Sperm Viability Kit (Molecular Probes). A membrane-permeable nucleic acid stain (SYBR14) labelled live sperm in green, and a membrane-impermeable stain (propidium iodide, PI) labelled dead sperm in red. Two microlitre of sample were diluted by the addition of 10 μL of ZSI, first stained with the addition of 1.5 μL SYBR14, incubated for 5 min at 36 °C and, then, stained with the addition of 1 μL of PI, followed by other 5 min of incubation at 36 °C. Five microlitre of stained sample were deposited on a slide, gently covered with a coverslip, and examined under a fluorescence microscope (Leica M165FC dissecting microscope equipped with a DFC7000T camera, Leica Microsystems) at ×400 magnification. The proportion of live sperm was calculated for at least 100 spermatozoa per slide, and the mean values of two slides were used for the analyses (within sample repeatability = 0.87 ± 0.06 st. error, see ref. ^22^.

### Sperm velocity

Sperm were activated by gently mixing with a Gilson micropipette 1 μL of ejaculate preserved in ZSI with 10 μL of aged tap water. Three microliters of samples were then quickly placed in separate wells on a 12-well multitest slide (MP Biomedicals) and covered with a 18 × 18 mm coverslip. Both slide and coverslip were previously coated with 1% polyvinyl alcohol (Sigma–Aldrich), to avoid sperm sticking to the glass slide^21^. Sperm velocity was measured using a CEROS Sperm Tracker (Hamilton Thorne Research) in the first 10 seconds after activation. The measurement was repeated on three subsamples per male, leading to a repeatability of 0.68 ± 0.14^22^, and mean values were used for statistical analyses. Mean velocity measurements were based on 280 ± 169 (mean ± s.d.) sperm tracks per male. Since sperm velocity parameters (VAP: average path velocity, VSL: straight-line velocity, and VCL: curvilinear velocity) were highly correlated (all Pearson R > 0.88; all *p* < 0.001), only VAP was considered in the following analyses. The measurement was repeated twice per male, yielding a repeatability of 0.68 ± 0.14^22^, and mean values were used for statistical analyses.

### Ethidium bromide and CLO treatments

Ethidium bromide treatment, to induce mtDNA depletion, was performed according to Rahn and colleagues^6^. Exposure to CLO (Clofilium tosylate, C2365, Sigma–Aldrich) was performed by incubating zebrafish larvae for 7 days (from 5 dpf to 12 dpf) in fish water-diluted drug, without methylene blue.

### Measurement of oxygen consumption rate (OCR)

OCR was measured on zebrafish embryos at 96-h post-fertilization (hpf) with a Seahorse XF24 extracellular flux analyser. Embryos were placed into a XF24 microplate well (1 embryo per well) and blocked with a capture screen in the presence of 670 μL of fish water (0.5 mM NaH_2_PO_4_, 0.5 mM NaHPO_4_, 3 mg/L instant ocean). The basal respiration was measured for 68 minutes at 28.5 °C. Respiratory rates are average ±SEM of at least 20 individual embryos per condition.

### Measurement of respiratory complex I activity

To measure the enzymatic activity of respiratory Complex I tissues were homogenized in a buffer composed of KH_2_PO_4_ at pH 7.4 in the presence of protease and phosphatase inhibitors. Total lysates were then quantified using a BCA Protein Assay Kit (Thermo-Scientific). Tissue homogenates were incubated for 5 minutes at 37 °C in a buffer composed of KH_2_PO_4_ pH 7.4, alamethicin 4 μM, BSA 6 mg/mL, sodium azide 1 mM, Coenzyme Q1 13.5 μM. Reactions were performed at 37 °C and started after the addition of the substrate NADH (60 μM). Complex I activity was measured following NADH oxidation at 340 nm (ε = 6.4 nM^−1^ cm^−1^) and subtracting the rotenone (20 μM) sensible fraction.

### Analysis of oxidative stress in zebrafish embryos

The MitoSOX indicator (M36008, Invitrogen, Thermo Fisher Scientific), was diluted to 5 mM in DMSO to make a reagent stock solution, as described in Mugoni and colleagues^23^. The stock solution was dissolved in HBSS (Hanks’s Balanced Salt Solution), to prepare a 5 μM working solution, and pre-warmed at 28.5 °C. MitoSOX was then added to an equal volume of fresh water on embryos and incubated at 28.5 °C in the dark for 25 min. Embryos were then rinsed three times in fresh fish water medium before imaging. Oxidation of the MitoSOX indicator by mitochondrial superoxide produces a red fluorescence, acquirable by confocal microscopy (absorption/emission at 510/580 nm).

### Imaging

For live imaging, transgenic embryos and larvae were anesthetized with Tricaine 1X (0.16 mg/mL), embedded in 1% low-melting agarose, placed on a depression slide and analysed with a Leica M165FC dissecting microscope equipped with a DFC7000T camera (Leica Microsystems) or using a Nikon Eclipse 90i upright microscope in a confocal C2 system (Nikon Instruments). Transgenic fluorescence of *Tg(Hsa.Cox8a:MLS-EGFP)*^*ia301*^ in *polg* mutant background was visualized under a Leica M165FC dissecting microscope and then with Leica SP5 and SP8 confocal microscopes through ×63 immersion objectives. WISH-stained embryos and larvae were mounted in 87% glycerol in phosphate-buffered saline plus and observed under a Leica M165FC microscope, equipped with a DFC7000T digital camera or with Leica SP5 confocal microscope. Histological samples were photographed on a Leica DMR using a DFC7000T digital camera.

### Signal quantification and statistical analysis

Zebrafish experiments implied two-sided tests on continuous variables (anatomical dimensions, fluorescence intensities, cell numbers), varying around a mean value with a 15% standard deviation, and with at least a 30% increase/decrease of the values in mutated/treated samples, compared to controls. With a 1:1 ratio between controls and mutated/treated samples, alpha error = 0.05 and statistical power = 95%, the required size of the sample population implied six controls and six mutated/treated samples (*N* = 6 samples per condition; Sample Size Calculator, ClinCalc.com). Individuals with gross congenital malformations (usually around 5–10% in an egg clutch) were excluded from the analysis. Samples from multiple fish crosses were randomized among all conditions, to prevent background-related differences. Blinded documentations were performed to avoid observer bias.

Zebrafish experiments were performed in triplicate on at least ten individuals per condition, and at least six samples were measured per condition (*N* = 6 per condition). For signal quantification, fluorescent dots were counted with the “Measurements” option of the Volocity 6.0 software (Perkin Elmer) or by Fiji software as already described^24^. After background subtraction, the integrated density of the signal (sum of pixels in a fixed volume) was normalized to control and indicated in the charts as Relative Intensity (R.I.). Statistical analysis was performed in GraphPad Prism 8.0. The Shapiro–Wilk normality test was used to confirm the normality of the data. In case data passed normality test (alpha = 0.05), then a parametric test, such as unpaired *t*-test or ordinary one-way ANOVA (in case of multiple comparisons) was used. In case the data did not pass the normality test, a nonparametric test was used (Mann-Whitney test or Kruskal–Wallis test for multiple comparisons). To correct for multiple comparisons, either Tukey’s multiple comparisons test (for ordinary one-way ANOVA) or Dunn’s multiple comparisons test (for Kruskal–Wallis test) was used.

The tests of sperm concentration, viability and velocity were performed using RStudio version 1.1.447. Data on sperm concentration and velocity were log transformed; data on sperm viability were arcsine transformed. Differences in sperm concentration, viability and velocity as an effect of male line (*polg*^*+/+*^ *vs. polg*^*sa9574/sa9574*^) were analysed by linear models (LMs). All LMs yielded normally distributed residuals (Shapiro–Wilk normality test. Sperm concentration: W = 0.940, *p* = 0.219; viability: W = 0.919; *p* = 0.125; velocity: W = 0.943, *p* = 0.360).

The values reported in the figures represent mean ± SEM calculated from at least six measures per condition. Significant differences from controls are indicated by asterisks. Correspondence between asterisks and significance levels is indicated in the figure legends.

## Results

### Knockdown of zebrafish *polg* affects embryonic growth, mitochondrial morphology and cell signalling

**Fig. 1:**
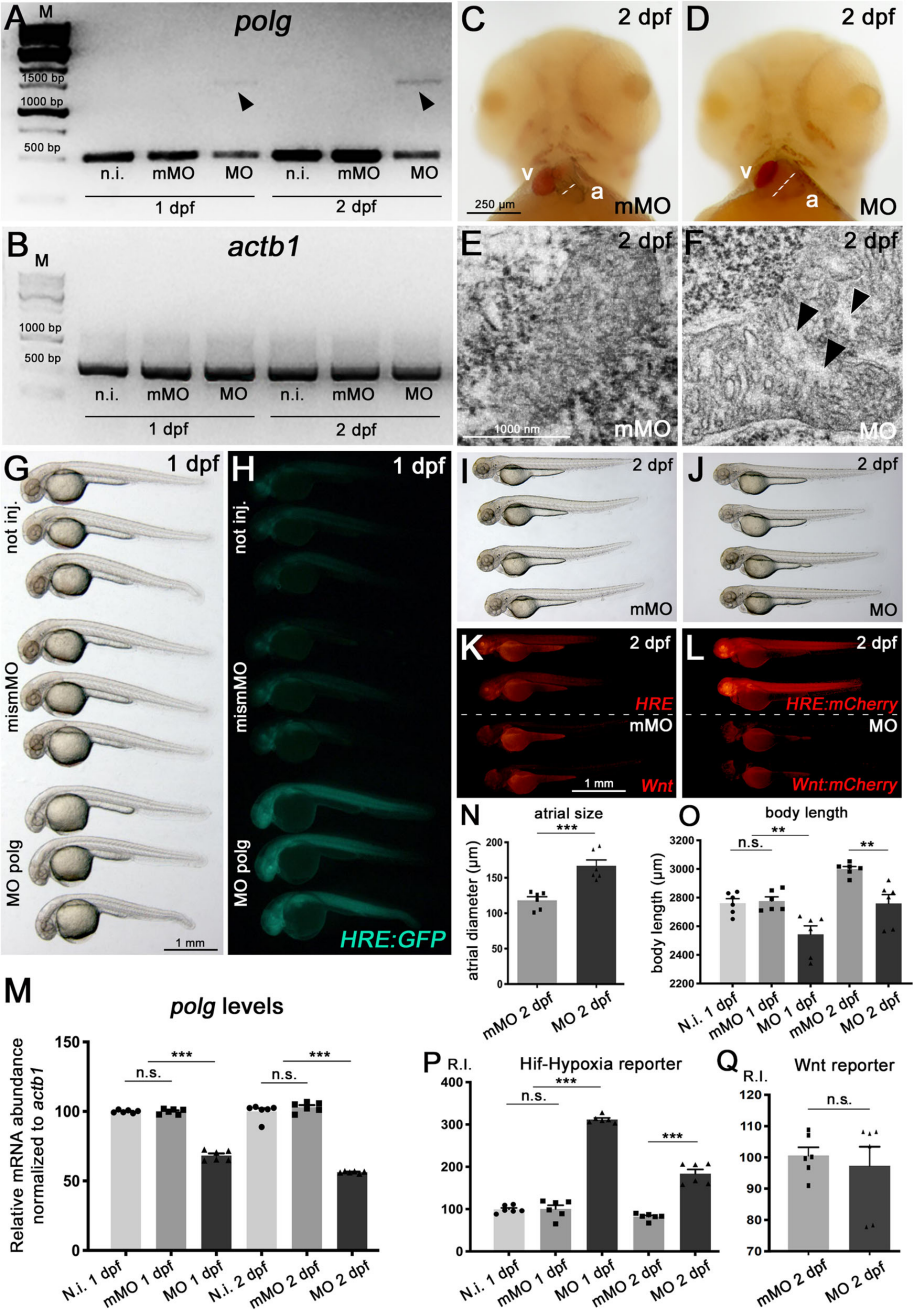
po*lg* morphants characterization and specific signalling pathway alteration. **A**, **B** Molecular validation of *polg-*specific morpholino oligos. Semi-quantitative RT-PCR from total cDNA of not injected (not inj), mismatched-morpholino (mismMO) injected controls and *polg*-specific morphants (MO), at 1 and 2 dpf, detects-specific alteration of *polg* transcripts. Arrowheads indicate altered cDNA products exclusively in *polg* morphant embryos (**A**). Control *actb1* transcripts appear unaltered under all conditions (**B**). M marker. PCR experiments were performed in triplicate on cDNA samples derived from *N* = 30 whole embryos per condition. Quantifications are displayed in the (**M**) chart. **C**, **D** Cardiac dilation in *polg* morphants. Frontal views of control (**C**) and polg morphant (**D**) embryos at 2 dpf. Note the enlarged atrium in the morphant; a atrium, v ventricle. Atrial measurements are displayed in the (**N**) chart. **E**, **F** TEM analysis of mitochondria in the skeletal muscle of 2-dpf embryos: normal mitochondrial morphology in controls (**E**); fused and swollen mitochondria in morphants (altered areas indicated by black arrowheads (**F**). **G**–**L** Analysis of Hif-Hypoxia and Wnt signalling pathways under *polg* deficiency. Bright field (**G**, **I**, **J**) imaging shows a decreased body length, in the absence of overt dysmorphology, in 1 and 2-dpf morphant embryos; measurements are displayed in the (**O**) chart. Fluorescence (**H**, **K**, **L**) imaging of Hif-Hypoxia (HRE Hif-Hypoxia Responsive Elements) and Wnt signalling reporters shows strong activation of the Hif-Hypoxia pathway under *polg*-deficieny, in both green (**H**) and red (**K**, **L**) transgenic background. On the contrary, Wnt signalling appears unchanged (compare **K** with **L**). Relative intensity (R.I.) quantifications of the fluorescent signals are displayed in the (**P**) and (**Q**) charts. Statistical tests: one-way ANOVA followed by Tukey’s test; ***p < 0.0001; **p < 0.001; n.s. not significant; *N* = 6 samples per condition, in triplicate.

To preliminarily establish the effects of a decreased activity of the *polg* gene in zebrafish, we transiently targeted the maturation of its transcript using splice-blocking morpholino oligomers. This treatment elicited the appearance of altered transcript forms and the halving of *polg* mRNA levels (Fig. 1A, B, M). Morphant embryos, examined within 3 dpf, displayed a reduced body size in the absence of gross dysmorphology (Fig. 1G, I, J, O), except for a peculiar enlargement of the cardiac atrium (Fig. 1C, D, N), associated with increased heart rate (shown later, in Fig. 6). Ultrastructural TEM analysis, performed in the skeletal muscle of morphant embryos, detected mitochondria frequently fused and with a swollen morphology (Fig. 1E, F).

Taking advantage of pathway reporter zebrafish lines, we could assess in vivo, at the whole-animal level, if *polg* deficiency affects metabolic signalling cascades relevant in mitochondria-to-nucleus cross-talk, such as Hif-Hypoxia and CREB pathways. We found that both Hif-Hypoxia signalling, verified with two independent reporters (Fig. 1H, K, L, P) and CREB signalling (Supplementary Fig. 1A, B, C) were upregulated in *polg* morphant embryos; a patterning pathway such as Wnt, also considered in the analysis, appeared unmodified at the same stages (Fig. 1K, L, Q). Collectively, these data suggest that *polg* knockdown, induced by antisense strategy, can affect embryonic metabolism, leading to reduced embryonic growth, mitochondrial dysmorphology and alteration of pathways involved in mitochondria-nucleus retrograde signalling.

### Genome editing of the *polg* gene; genotyping of *polg*^*ia302*^ and *polg*^*sa9574*^ mutant lines

The zebrafish Polg protein contains 1206 amino acids (aa) and is composed of three major domains: exonuclease domain (230–421), linker region (421–741) and polymerase domain (741–1167). Zebrafish Polg has the same functional domains and a 69% overall identity (79% similarity) to human POLG^18^.

In 2015 Rahn and colleagues generated a zebrafish mutant for the polymerase domain of the *polg* gene, characterized by a rapid and sustained mitochondrial DNA depletion, altered energetics and growth, which failed to survive to 4 weeks^6^.

To generate a new zebrafish model, completely devoid of polymerase gamma activity, our group mutated the zebrafish *polg* gene in the exonuclease domain, using the CRISPR/Cas9 approach.

**Fig. 2:**
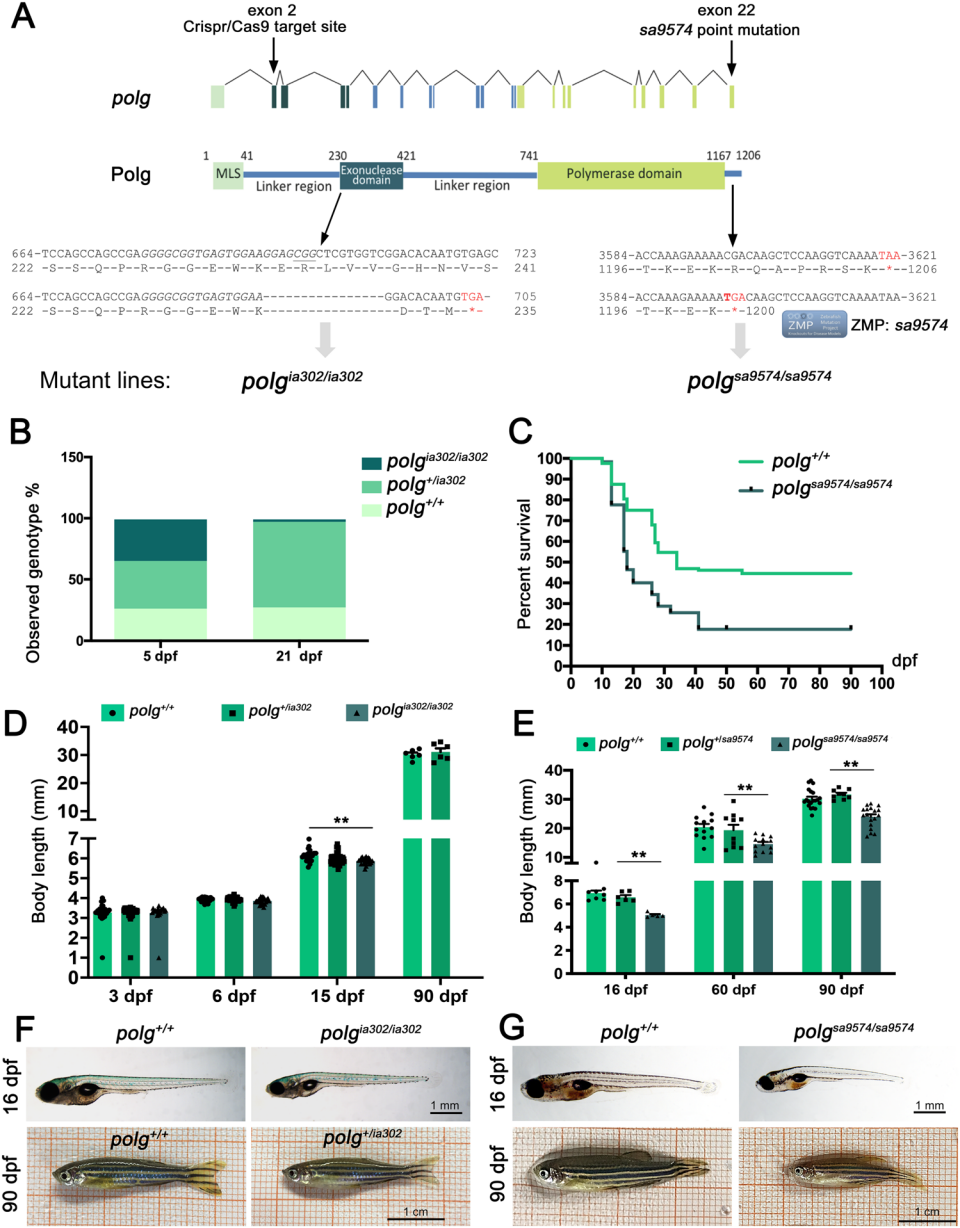
Editing of the zebrafish *polg* gene. **A** Zebrafish *polg* exons are shown as color-coded boxes corresponding to protein domains of the Polg peptide. Black arrows show the position of the CRISPR/Cas9-targeted site in exon 2 of the *polg*^*ia302*^ mutant line, and the missense mutation in exon 22 of the *polg*^sa9574^ mutant line. In the *polg*^*+/+*^ (up) vs. *polg*^*−/−*^ (down) sequences, the CRISPR/Cas9-targeted site is in italic and the protospacer adjacent motif (PAM) is underlined. The new stop codons generated in both mutants are indicated with * = TGA, in red. **B**, **C** *polg*^*ia302/ia302*^ individuals fail to survive to three weeks (**B**), while part of *polg*^*sa9574/9574*^ animals can survive to adulthood (**C**). In the Kaplan–Meier survival curve for *polg*^*sa9574*^, time is shown in days post-fertilization (dpf); *N* = 50 individuals per genotype. The Log Rank test was used for statistical analysis; *polg*^*+/+*^ vs. *polg*^*sa9574/sa9574*^: *p* < 0.0001. **D** Body length analysis in wt *(polg*^+/+^), heterozygous (*polg*^*+/ia302*^) and homozygous (*polg*^*ia302/ia302*^) individuals. The body length of zebrafish *polg*^*ia302/ia302*^ mutants is significantly reduced compared to controls at 15 dpf. Heterozygotes body length is indistinguishable from wt at all considered stages. *N* = 8, in triplicate; **p < 0.01. **E** Body length analysis in wt *(polg*^+/+^), heterozygous (*polg*^+/sa9574^) and homozygous (*polg*^*sa9574/sa9574*^) individuals. The body length of zebrafish *polg*^sa9574/sa9574^ mutants is significantly reduced compared to control and heterozygous individuals at all considered stages. *N* = 8, in triplicate; *p < 0.05; **p < 0.01. **F** Representative images of a zebrafish *polg*^*+/+*^ control and a *polg*^*ia302/ia302*^ mutant at 16 dpf; a *polg*^*+/+*^ control is compared with a *polg*^*+/ia302*^ heterozygote at 90 dpf. **G** Representative images of a zebrafish *polg*^*+/+*^ control and a *polg*^*sa9574/sa9574*^ mutant at 16 and 90 dpf.

A heterozygous F1 offspring with a 16-nucleotide deletion in exon 2 (allele *polg*^*ia302*^) was selected and used to obtain the F2 generation (Fig. 2A). All genotypes could be easily distinguished by PCR amplification and agarose gel analysis of DNA obtained by fin clips (Supplementary Fig. 2A). The 16-nucleotide deletion resulted in a frameshift mutation that leads to a 3-aa miscoded protein sequence after aa 231, and a premature stop at aa 235. F2 individuals were sequenced to confirm that they shared an identical mutation (Supplementary Fig. 2B).

In addition, we obtained from the European Zebrafish Resource Centre (EZRC) a potentially hypomorphic mutant called *polg*^*sa9574/sa9574*^, bearing a rather terminally located C/T transition that leads to a nonsense mutation, with the loss of the last 6 aa (Fig. 2A). Genotyping was performed by PCR combined with high-resolution melting analysis (HRMA). This approach is rapid, sensitive, and cost-effective, with low risk of contamination artefacts (Supplementary Fig. 2C). Sequencing of F2 individuals fully confirmed the presence of the expected *sa9574* point mutation (Supplementary Fig. 2D).

### The *polg*^*ia302*^ and *polg*^*sa9574*^ mutant lines display different survival rates

We next investigated the survival rate for all genotypes in both mutant lines. Concerning the *polg*^*ia302*^ line, the observed genotypes in the progeny from two heterozygous fish did not significantly differ from the expected proportions (25% *polg*^*+/+*^, 50% *polg*^*+/ia302*^ and 25% *polg*^*ia302/ia302*^), when analysed at 5 dpf. However, this distribution was significantly different from the expected ratios at 21 dpf (Fig. 2B). These data indicate that *polg*^*ia302/ia302*^ animals have normal survival at early larval stages, but begin to die at later stages and, similarly to what observed for *polg* mutants generated by Rahn and colleagues^6^, homozygous mutants cannot reach adulthood. Since adult *polg*^*ia302/ia302*^ are not viable, most of the experiments shown in this paper were obtained from the *polg*^*sa9574/sa9574*^ line.

Survival of the *polg*^*sa9574*^ line was determined by collecting control and homozygous mutant embryos, and growing them until 3 months. This analysis showed that *polg*^*sa9574/sa9574*^ animals have normal survival until 2 wpf (weeks post-fertilization) but begin to die after that time. The critical rearing period for all genotypes was found between 10 and 25 dpf. Thereafter, the decline in the survival rate levelled at around 45% in wt fish and 18% in *polg*^*sa9574/sa9574*^ mutants (Fig. 2C). This decrease in early life survival was statistically significant comparing *polg*^*+/+*^ with *polg*^*sa9574/sa9574*^.

### Homozygous *polg*^*ia302*^ and *polg*^*sa9574*^ mutants display a reduced body size at larval and adult stages

We quantified the growth characteristics of wt, *polg*^*ia302*^ and *polg*^*sa9574*^ animals during the first three months of development. Figure 2D, E, from juvenile to adult stages (15–16 to 90 dpf), highlights the reduced body development of mutant individuals compared to wt. The body length (measured from the snout to the base of the caudal fin) is a quantifiable indicator of development in zebrafish. The measurements were performed on wt, heterozygous and homozygous fish for the mutation. Homozygous mutants grew more slowly than wt and were significantly smaller than control siblings from the juvenile stage onwards (Fig. 2D–G). This reduced growth is maintained in *polg*^*sa9574/sa9574*^ mutants until fish adulthood (Fig. 2E, G).

### Multiorgan defects and reproductive impairment in *polg*^*sa9574*^ mutants

Survival during adulthood of the hypomorphic *polg*^*sa9574*^ mutant line could allow us to evaluate *polg* mRNA levels in a tissue-specific manner. This analysis detected a general decrease of *polg* expression in all considered tissues, especially in brain, eye and heart (Supplementary Fig. 2E).

**Fig. 3:**
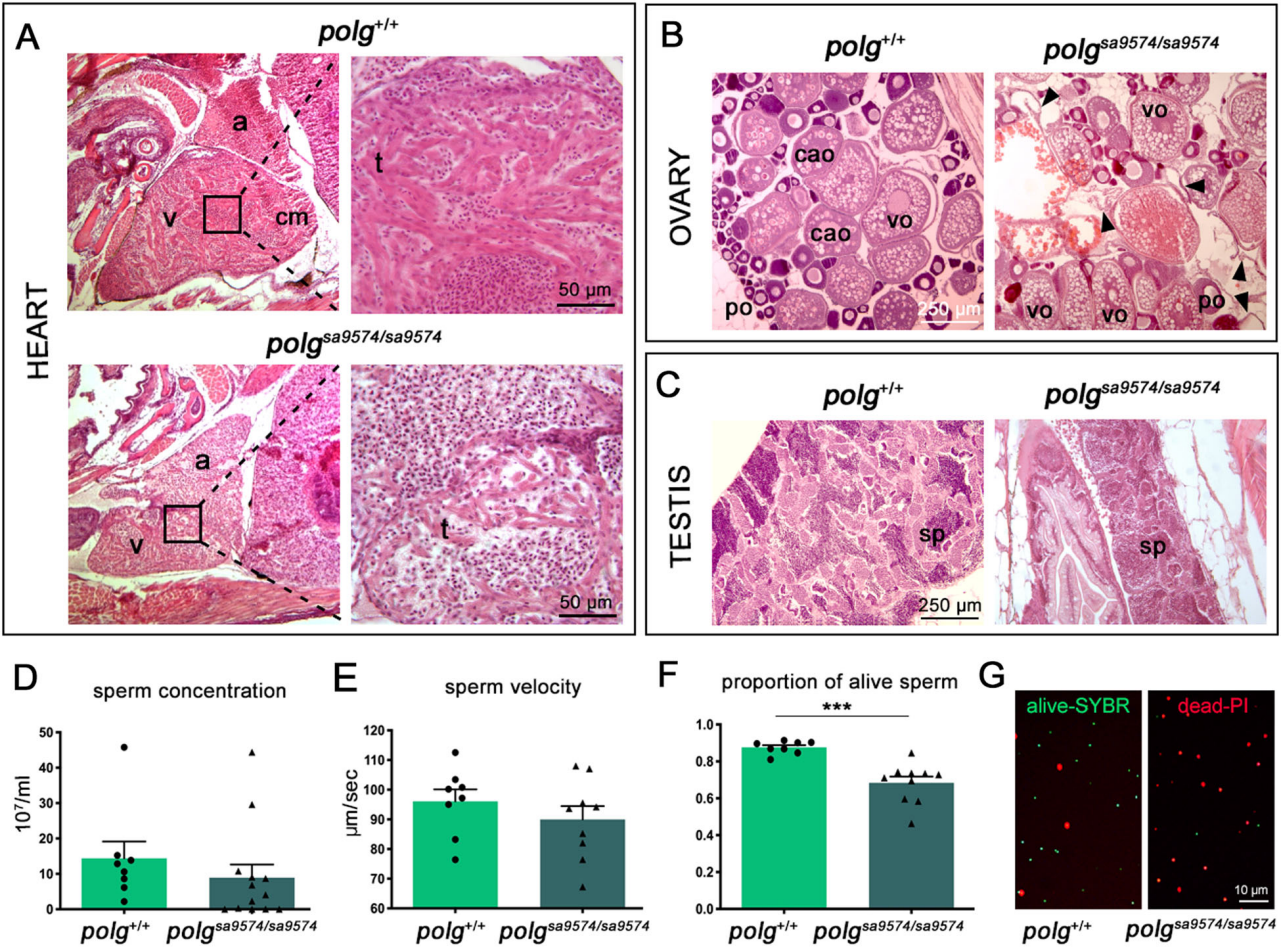
Effects of Polg mutation on heart and gonads of adult zebrafish. **A** Cardiac EE histology shows a clear reduction of the thickness of the trabecular network in mutant hearts; a atrium, cm compact myocardium, t trabeculae, v ventricle. **B** Ovarian EE histology detects oocyte maturation defects in mutant females; black arrowheads indicate debris of atretic follicles. po primary oocyte, cao cortical-alveolar oocyte, vo vitellogenic oocyte. **C** Testicular EE histology detects a reduced amount of spermatozoa (sp) in mutant males. Concentration (**D**), velocity (**E**) and viability (**F**) of ejaculated sperm. Depicted is mean ± SEM. ***p < 0.001; *N* = 10, in triplicate. **G** Illustrative image of fluorescence staining evidencing a higher amount of dead (red) over alive (green) sperm in *polg*^*sa9574/sa9574*^ males.

Cardiac histology on 4-month post-fertilization (mpf) *polg*^*sa9574/sa9574*^mutants showed dilated heart and reduction of the trabecular network and its thickness, compared to controls (Fig. 3A). Gonadal histology was also performed, detecting anomalies in both sexes. Wild-type females at 5 mpf presented normal ovaries, with follicles at all developmental stages, ranging from primary to full growth stage (Fig. 3B). Histological analysis of age-matched *polg*^*sa9574/sa9574*^ females showed instead an increase of atretic oocytes and subsequent debris, with interruption and fragmentation of the vitelline envelope, suggesting alteration of follicle maturation (Fig. 3B). As far as concerns male gonads, spermatozoa at all developmental stages were detected in both wt and *polg*^*sa9574/sa9574*^ testes; however, mutant testes were comparatively smaller and with a reduced amount of sperm (Fig. 3C).

As we noted that *polg*^*sa9574*^ mutants had difficulties to reproduce, we focused our attention on sperm quality. Sperm concentration did not significantly differ between *polg*^*+/+*^ and *polg*^*sa9574/sa9574*^ males, although a trend to a lower sperm concentration in *polg*^*sa9574/sa9574*^ was observed (*p* = 0.079) (Fig. 3D). Moreover, there was no difference in sperm velocity between *polg*^*+/+*^ and *polg*^*sa9574/sa9574*^ ejaculates (LM: estimate = 0.070, st.err. = 0.070, *t*-value = 1.013, *p* = 0.327, d.f. = 15) (Fig. 3E). However, ejaculates from *polg*^*sa9574/sa9574*^ males contained a significantly lower amount of alive sperm compared to controls, as revealed by SYBR-green/PI assay (LM: estimate = 0.309, st.err. = 0.056, *t*-value = 5.483, *p* < 0.001, d.f. = 16) (Fig. 3F, G).

**Fig. 4:**
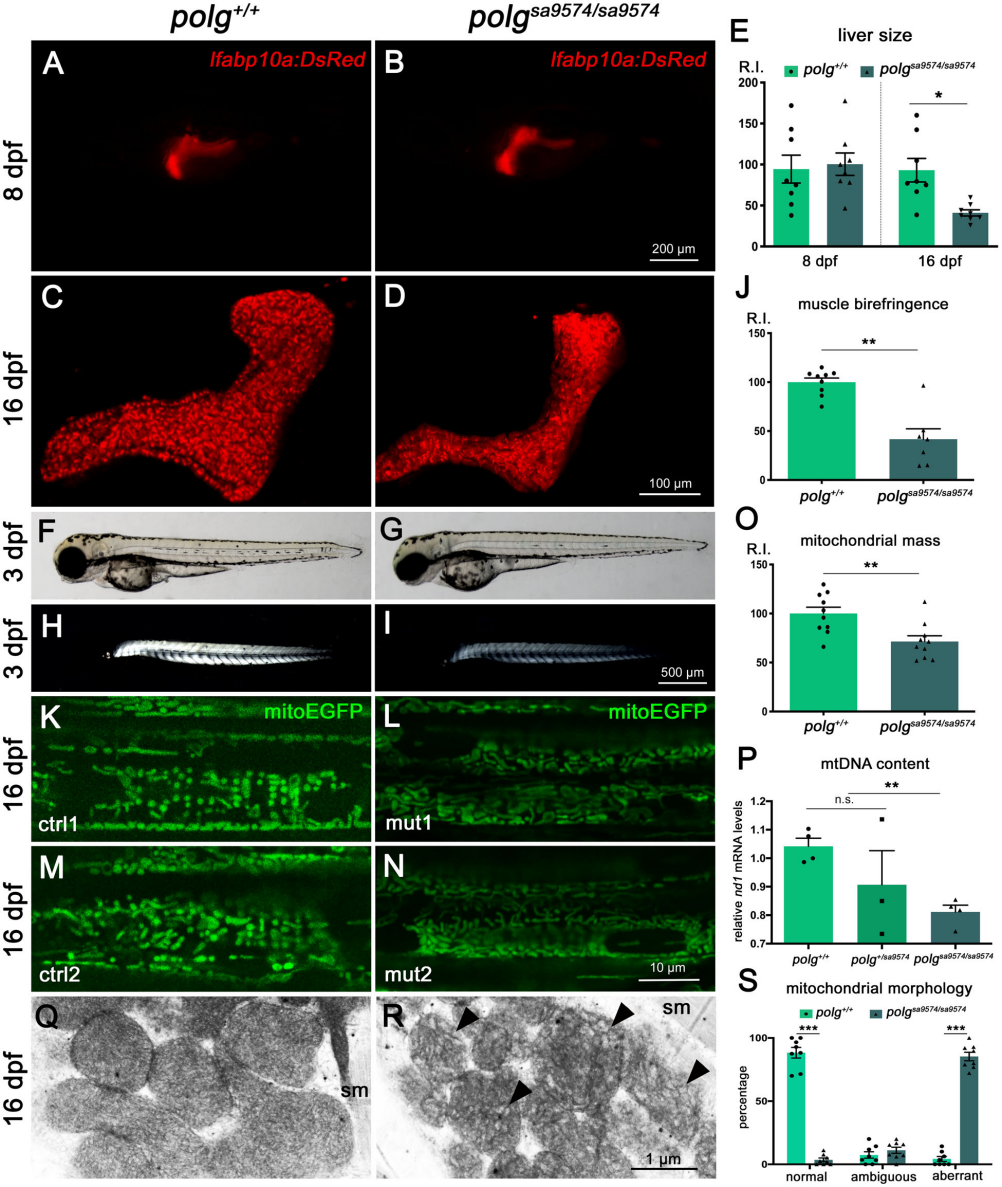
Effects of Polg mutation on liver, skeletal muscle and mitochondria. **A**–**D** Representative Z projections of 3D confocal images of wt and *polg*^*sa9574/sa9574*^ liver at 8 and 16 dpf. The quantification of liver-specific fluorescence from the *lfabp10a:DsRed* transgene (**E**) indicates reduced liver size in 16 dpf mutants. **F**–**I** Reference bright field images (**F**, **G**) and light microscopy analysis of muscle birefringence (**H**, **I**) in wt and *polg*^*sa9574/sa9574*^ embryos at 3 dpf. The birefringence quantification (see Methods) shows reduced signal in mutants (**J**). **K**–**N** Confocal images of the *Tg(Hsa.Cox8a:MLS-EGFP)*^*ia301*^ mitochondrial marker (mitoEGFP) in wt (**K**, **M**) and *polg*^*sa9574/sa9574*^ (**L**, **N**) at 16 dpf. The relative quantification of integrated density (**O**) indicates a reduced mitochondrial mass. **P** The relative quantification of mtDNA copy number, in wt and *polg sa9574* mutants, shows a significant reduction of mtDNA in homozygotes. Data are presented as mean ± SEM and were generated from three different experiments, each containing six embryos per genotype and treatment condition (*p < 0.05; **p < 0.01; *N* = 6, in triplicate). **Q**, **R** TEM analysis of mitochondria in 16 dpf skeletal muscle fibres (sm) of wt and *polg*^*sa9574/sa9574*^ detects vesicular cristae with “honeycomb” arrangement inside the mutated mitochondria (indicated by black arrowheads). **S** Quantification of aberrant morphologies; ***p < 0.001; *N* = 8 independent TEM images per condition, in triplicate.

Finally, taking advantage of fluorescent transgenes and birefringence properties in the zebrafish organism, we investigated the morphology of liver and skeletal muscle, two of the most affected tissues in Polg-related disease^25^. For liver analysis, we measured the fluorescence of liver-specific transgenic zebrafish at 8 and 16 dpf (Fig. 4A–D). The statistical analysis did not reveal any significant difference between 8 dpf *polg*^*+/+*^ and *polg*^*sa9574/sa9574*^ larvae, while a decreased signal, connected to a reduced (nonisometric) organ size, was found in mutants at 16 dpf (Fig. 4E).

To assess for in vivo defects in the amount and/or organization of myofibrils, we exploited the birefringence properties of zebrafish skeletal muscle. The analysis, performed in 3 dpf embryos, revealed similar signals in control and heterozygous embryos (not shown), while a strong birefringence decrease was detected in *polg*^*sa9574/sa9574*^ larvae compared to wt (Fig. 4F–J).

In summary, these findings indicate the presence of significant multiorgan defects in *polg*^*sa9574*^ mutant larvae and adults, involving liver size, skeletal muscle mass, heart morphology and gonadal function, the latter characterized, in males, by reduced viability of the mutant sperm.

### *polg*^*sa9574*^ mutants display alterations in mitochondrial mass, organization, function and retrograde signalling

The multisystem defects detected in *polg* mutants corroborate the important role of Polg activity and mitochondrial integrity in the maintenance of organ size and function. Previous research, for instance, has suggested that sarcopenia correlates with a loss of mitochondria content^1,26^. To assess the mitochondrial organization in the skeletal muscle of *polg*^*sa9574*^ mutants, we took advantage of a mitochondrial-targeted transgene *Tg(Hsa.Cox8a:MLS-EGFP)*^*ia301*^ (“mitoEGFP”) allowing the fluorescent visualization of these organelles in vivo. With this tool, we could detect an alteration of organelle distribution along the fibres and a decrease of mitochondrial mass (Fig. 4K–O), which was accompanied by a significant reduction of mtDNA content in homozygous mutants, as assessed by qPCR (Fig. 4P). In parallel, transmission electron microscopy analysis could identify a significant increase of mitochondria with aberrant cristae morphology in the skeletal muscle of *polg*^*sa9574/sa9574*^ mutants (Fig. 4Q–S).

**Fig. 5:**
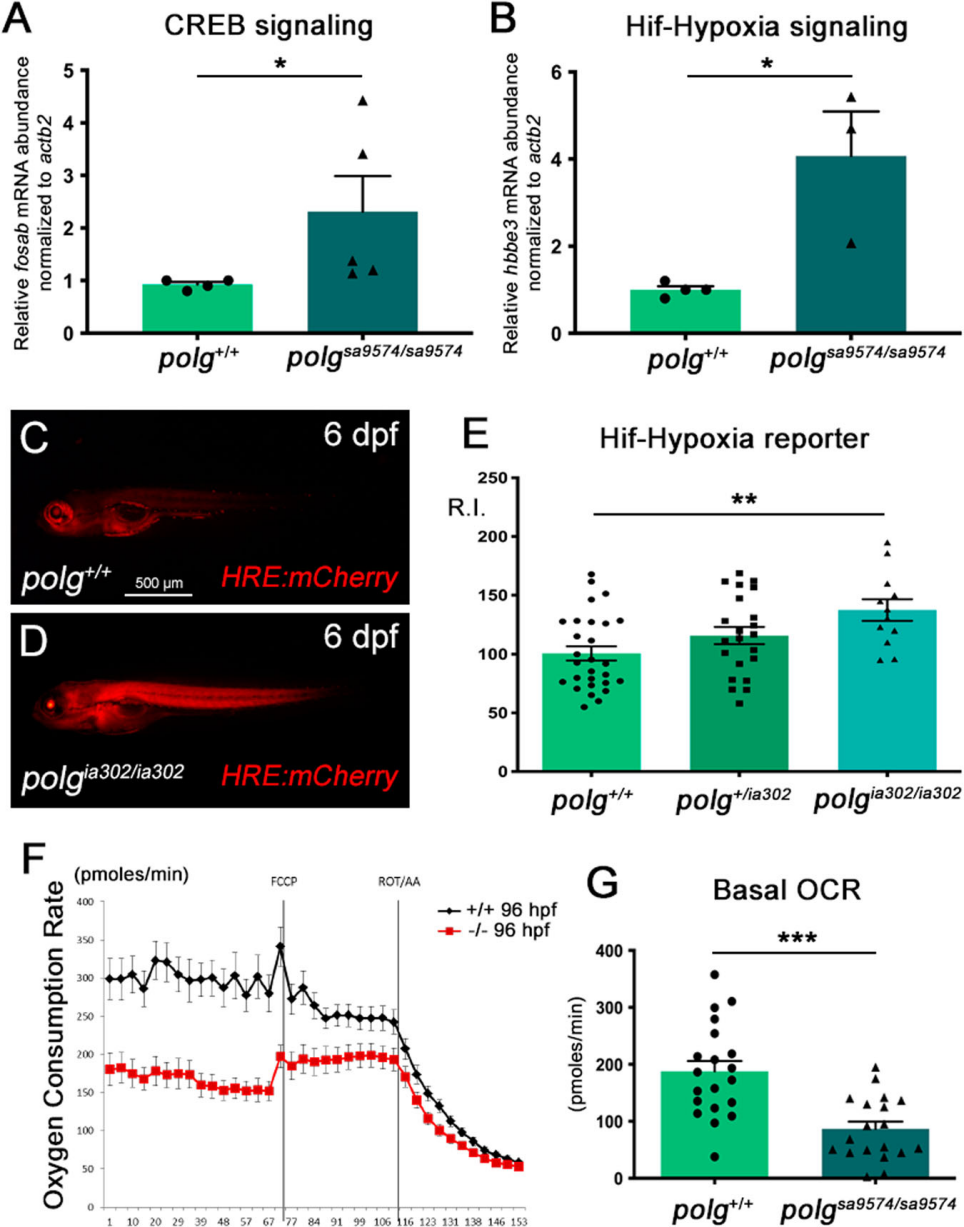
Altered signalling and mitochondrial dysfunction in *polg* mutants. **A** The CREB signalling-targeted gene *fosab* is upregulated in 4 dpf *polg*^*sa9574/sa9574*^ mutants, compared to controls. **B** The Hif-Hypoxia signalling-targeted gene *hbbe3* is upregulated in 4 dpf *polg*^*sa9574/sa95*^ mutants, compared to controls. **C**–**E** Hif-Hypoxia signalling increases also in 6-dpf *polg*^*ia302/ia302*^ mutants, as shown by the Hif-Hypoxia-responsive transgene *HRE:mCherry*. **E** Chart reporting the HRE fluorescence relative intensity (R.I.). **F** Diagram depicting the oxygen consumption rate (OCR) profile throughout Seahorse assay in 4 dpf wt and *polg*^*sa9574/sa9574*^ embryos under basal conditions, FCCP (p-triFluoromethoxyCarbonylCyanide Phenylhydrazone)-induced maximal respiratory capacity stimulation and ROT/AA (Rotenone/Antimycin A)-mediated inhibition. Three independent experiments were performed (*N* = 20). **G** Quantification of basal respiration in wt and *polg*^*sa9574/sa9574*^ mutants (*N* = 20, in triplicate).

Led by the results obtained with transient KD (Fig. 1), we double-checked if these mitochondrial phenotypes could arise in association with the stimulation of typical mitochondria-to-nucleus retrograde communications. When focusing our attention on CREB and Hif-Hypoxia signalling, we detected a 3–4-fold increase in the activation of both pathways in *polg*^*sa9574/sa9574*^ mutants (Fig. 5A, B). Notably, Hif-hypoxia hyper-activation was also confirmed in vivo in *polg*^*ia302*/ia302^ mutants, using a Hif-hypoxia-responsive transgene (Fig. 5C–E).

Finally, to verify more directly whether *polg* mutation could affect mitochondrial function, we measured the oxygen consumption rate (OCR) using a Seahorse extracellular flux analyser. The oxidative respiration was analysed in 4 dpf whole embryos, first under basal conditions, then under FCCP stimulation and, finally, under Rotenone/Antimycin A-mediated inhibition, which allows the evaluation of nonmitochondrial oxygen consumption (Fig. 5F). The G chart in Fig. 5 compares wt and mutant net mitochondrial respiration. Quantifications of these measurements show a highly significant decrease of basal OCR in *polg*^*sa9574/sa957*^ homozygotes, compared to controls, confirming the mitochondrial dysfunctionality in this mutant line.

During oxidative phosphorylation in the mitochondrion, molecular oxygen is transformed into highly reactive oxygen species (ROS) in the electron transport chain. Taking advantage of the MitoSOX indicator for in vivo visualization of mitochondrial superoxide production, we observed a decrease of the signal in *polg*^*sa9574/sa957*^mutants compared to controls (Supplementary Fig. 3a–c). In line with this observation, *polg*^*sa9574/sa957*^mutants also showed decreased expression of ROS-induced genes *nqo1* and *txnrd3* (Supplementary Fig. 3D), further corroborating a reduced mitochondrial activity in mutants.

### *Polg*^*ia302*^ mutants display alterations in myofibrils, locomotion, ROS and retrograde signalling

Due to their larval lethality, *polg*^*ia302/ia302*^ mutants have been only preliminarily characterized, within a 2-week time frame; specifically, reduced birefringence was detected at 3 dpf in homozygous individuals, compared to heterozygous and wt siblings, suggesting early myofibril disorganization (Supplementary Fig. 4A, A’). To further investigate this aspect, locomotion tests were performed on all genotypes, at 6 and 15 dpf. Behavioural assays showed that the genetic ablation of *polg* does not affect the normal responses to light stimuli of 6-dpf larvae (Supplementary Fig. 4B, B’) However, at 15 dpf, both heterozygous and homozygous larvae display altered responsiveness to light stimuli, as the total distance swum by these individuals appeared significantly reduced compared to wt siblings (Supplementary Fig. 4C, C’). These results demonstrate that *polg* mutations may affect locomotor ability of zebrafish larvae, and that this effect can be appreciated also under heterozygous condition. Further characterizations have been performed in *polg*^*+/ia302*^ heterozygotes during adulthood, failing to detect significant differences in mtDNA content within selected organs, such as heart, brain, gonads and skeletal muscle (Supplementary Fig. 4D). Interestingly, though, heterozygous *polg*^*+/ia302*^ individuals showed increased expression of Hif-Hypoxia and CREB targets, and reduced expression of ROS-induced genes, at both cardiac and brain level (Supplementary Fig. 4E, F), strikingly in line with observed variations in *polg* morphants and *polg*^*sa9574*^ mutants, suggesting that this retrograde signalling pattern could represent a common molecular signature under Polg dysfunction.

### CLO treatment can rescue Polg-related mitochondrial and cardio-skeletal muscle defects

As the Clofilium tosylate (CLO) compound has been shown to rescue Polg-related phenotypes in yeast and nematode^8^, we tested its effects at the whole vertebrate animal level, exploiting our zebrafish *polg* mutants. First, to determine the optimal working dose, wt individuals were grown with increasing concentrations of CLO, from 5 dpf (mouth opening stage) up to 12 dpf. The drug was directly diluted in fish water. Supplementary Fig. 5A represents the survival chart. Among the tested doses, the highest non-toxic concentration of CLO was 5 μM, while higher concentrations resulted in toxic effects. Indeed, larvae exposed to 10 µM CLO displayed malformations in the pectoral fin, reduced growth, curved body and microcephaly (Supplementary Fig. 5B–E).

**Fig. 6:**
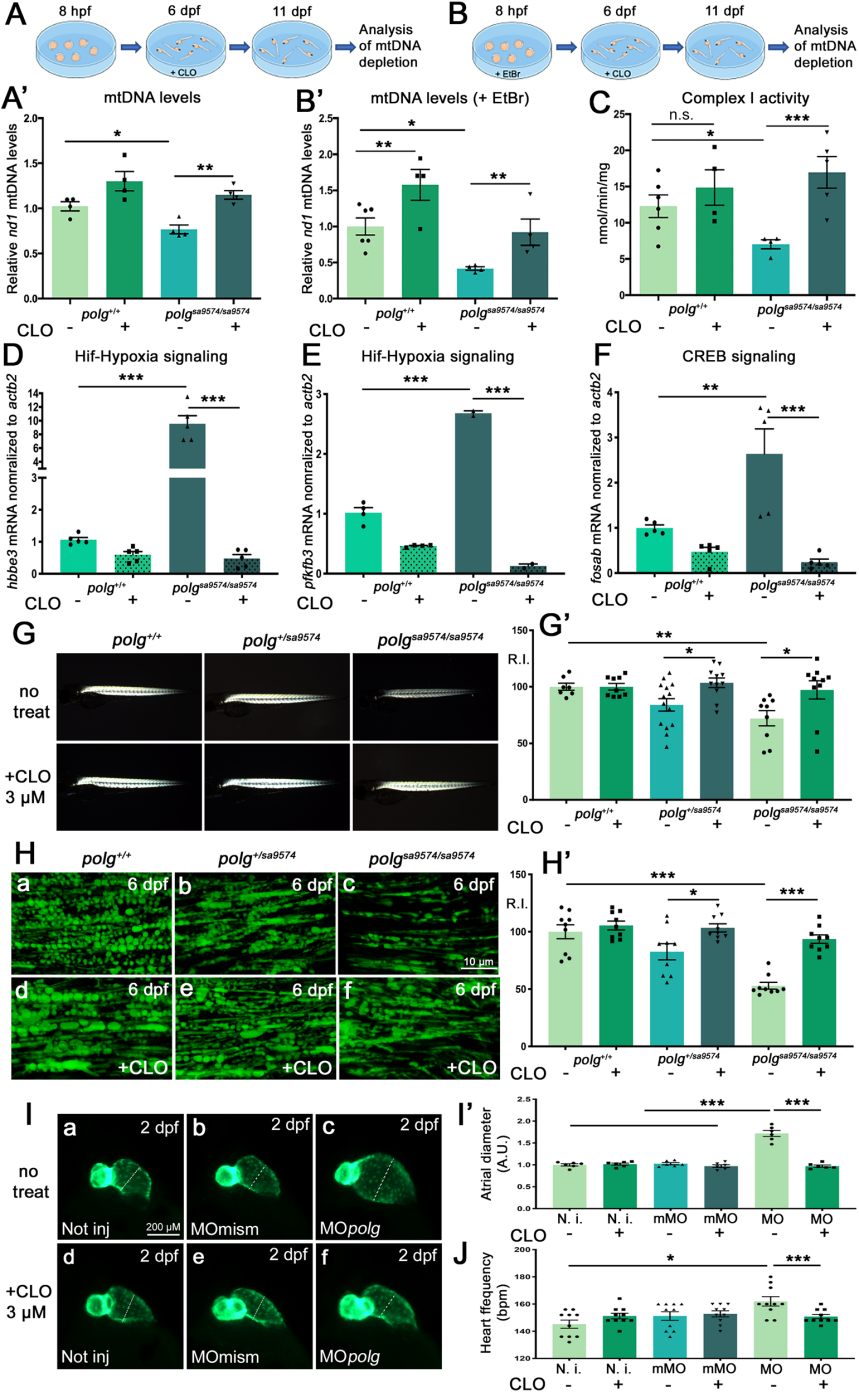
The CLO compound can rescue mitochondrial and cardio-skeletal defects in *polg* mutants. **A**, **B** Comparison of relative abundances of the mitochondrial *nd1* gene in the presence of CLO, in wt and *polg*^*sa9574/sa9574*^ zebrafish, untreated (**A**) or treated (**B**) with Ethidium Bromide (EtBr). *polg*^+/+^ zebrafish recover from EtBr-induced mtDNA depletion while *polg*^*sa9574/sa9574*^ mutants remain depleted of mtDNA. (**A**’, **B**’) Data in **A**’ and **B**’ charts were generated from three different experiments, each containing a pool of six embryos per genotype and treatment condition (*N* = 6, in triplicate). **C** Complex I activity in *polg*^*sa9574/sa9574*^ adult muscle tissue is significantly lowered, and can be rescued after CLO treatment. Data were generated from three different experiments, each containing a pool of four samples per genotype and treatment condition (*N* = 4, in triplicate). **D**–**F** Retrograde signalling decrease after CLO treatment, based on the expression of the Hif-Hypoxia targets *hbbe3* and *pfkfb3* (**D**, **E**), and the CREB-responsive gene *fosab* (**F**); *N* = 5. **G** Muscle birefringence, reduced in *polg*^*sa9574/sa9574*^ mutants compared to siblings, can be rescued by CLO treatment. **G**’ Relative intensity (R.I.) quantifications of experiments shown in **G**; *N* = 10 per genotype. **H** The mitochondrial mass, analysed at 6 dpf by mitochondria-targeted EGFP *Tg(Hsa.Cox8a:MLS-EGFP*) in the skeletal muscle of all *polg*^*sa9574*^ genotypes, is reduced in homozygotes (compare **C** with **A**, **B**), and can be rescued by CLO treatment (**D**–**F**). **H**’ Relative intensity (R.I.) quantifications of experiments shown in **H**; *N* = 9 per genotype. **I** The atrial diameter (dashed white lines), enlarged in 2-dpf *polg* morphants (**C**) compared to not injected (**A**) and control-injected (**B**) embryos, can be restored to normal values after CLO treatment (**D**–**F**). Cardiac chambers are visualized by *Tg(tg:EGFP,myl7:EGFP)*^*ia300*^. **I**’ Quantifications of experiments shown in **I**; the atrial diameter is expressed in arbitrary units (A.U.); *N* = 6 per condition. **J** The heart rate, increased in *polg* morphants (MO) compared to controls (N.i.; mMO), can be restored to normal values after CLO treatment; *N* = 10 per condition; Bpm beats per minute. All charts in Fig. 6 display data as mean ± SEM; n.s. not significant; *p < 0.05; **p < 0.01; ***p < 0.001.

Once the toxicity of CLO was determined, we proceeded to test the efficacy of this drug in relation to the mtDNA depletion phenotype. As previously shown (Fig. 4P), in the absence of treatment *polg*^*sa9574/sa9574*^ mutants displayed a 20–25% reduction of mtDNA, compared to healthy controls (see also Fig. 6A, A’). Addition of 5 μM CLO to fish water (from 6 to 11 dpf) induced an increase of mtDNA copy number in *polg*^*sa9574/sa9574*^ mutants, reaching a 1.5-fold change compared to the untreated mutants. Notably, CLO treatment of healthy controls could also induce a 1.3-fold increase of mtDNA, compared to the reference level (Fig. 6A, A’).

To obtain a stronger reduction of mtDNA in controls and *polg*^*sa9574/sa957*^ mutants, we performed an ethidium bromide (EtBr) treatment. EtBr is an intercalating dye that preferentially incorporates into circular DNA, halting replication and transcription. Treatment with 15 µg/mL EtBr for 5 days can significantly reduce the relative quantity of mtDNA in zebrafish larvae, compared to untreated controls^6^. In our setup, embryos were pre-treated with EtBr for the first 5 days of development, after which they were rinsed and allowed to grow in the presence or absence of CLO for 6 days. At 11 dpf, in the absence of CLO, *polg*^*sa9574/sa9574*^ animals exposed to EtBr had significantly lower mtDNA values (58.2% reduction) than *polg*^*+/+*^ treated fish, suggesting delayed or decreased mtDNA repopulation rates for the mutant Polg compared to the wt enzyme. At 11 dpf, all CLO-treated larvae had higher amounts of mtDNA than untreated individuals of the same genotype, with an increase by 1.6-fold in wt and 2.3-fold in mutants, compared to untreated controls (Fig. 6B, B’). This increase of mtDNA due to CLO treatment is significantly higher in animals pre-treated with EtBr, compared to those not exposed to EtBr, supporting the CLO treatment as a highly effective in vivo treatment for the restoration of mtDNA depletion in a Polg-deficient vertebrate system.

An additional Polg-related phenotype, associated to mtDNA depletion, is represented by the reduced amount of mitochondrial-encoded proteins, including Complex I components, which lead to typical Complex I deficiencies observed in Polg patients^27^. We have verified Complex I activity in mutants, detecting a 43% decrease of its efficiency in the skeletal muscle of adult mutants, compared to wt tissue (Fig. 6C). Consequently, we decided to investigate whether Complex I activity could be rescued by CLO treatment, in this case performed at the adult stage. The administration of CLO for 6 days on 6-mpf individuals could induce a 2.4-fold increase of Complex I performance in treated mutants, compared to untreated ones (Fig. 6C), supporting CLO as an effective drug for the rescue of Complex I activity under Polg deficiency, with proved efficacy also during adulthood. Given the net effect of CLO on mitochondrial function, we checked if this drug could affect the mitochondria-to-nucleus retrograde signalling, detecting a striking reduction of both Hif-Hypoxia- and CREB-mediated response (Fig. 6D–F). Finally, focusing on *polg*^*sa9574*^ skeletal muscle, we found that CLO treatment could successfully rescue the myofibril organization, analysed through birefringence (Fig. 6G, G’), and the mitochondrial mass, visualized by mitochondrial-directed EGFP (Fig. 6H, H’). Notably, also cardiac phenotypes such as atrial enlargement and tachycardia, induced by morpholino-mediated Polg deficiency, could be restored to normal values (Fig. 6I, J) after a 2-day treatment with CLO, supporting a multiorgan efficacy of this drug.

## Discussion

The current lack of satisfactory POLG models and of an effective therapy for POLG-related disorders is the main aspect that inspired our work. The use of different approaches to model POLG dysfunction in zebrafish (antisense oligomers, point mutants and deletion mutants) represents a powerful strategy that allowed us to compare the induced phenotypes under transient loss-of function, as well as under stable hypomorphic and severe gene disruption. Specifically, in this study, the robust comparison among different zebrafish POLG models took advantage of multiple techniques, including the *polg* downregulation by morpholino, and the use of two stably mutated *polg* alleles: an adult-viable ENU-induced point mutation (line *polg*^*sa9574*^) and a larval-lethal CRISPR/Cas9-induced microdeletion (line *polg*^*ia302*^).

Interestingly, under different conditions, we could note the exhibition of common phenotypes, including developmental delay, reduced body size, cardiac defects and mitochondrial dysmorphology. Of note, both Polg downregulation and stable mutation can activate mitochondria-to-nucleus retrograde signalling such as Hif-Hypoxia and CREB pathways, both representing adaptive responses that communicate to the nucleus the status of mitochondrial metabolism and genetic instability^28–30^. Specifically, CREB signalling can sense mitochondrial dysfunction that leads to increased intracellular calcium, while Hif-Hypoxia signalling, mediated by hypoxia-inducible factors (Hif), is activated by low O_2_ tensions but also under mitochondrial dysfunction, and/or accumulation of intracellular metabolites^31^, leading to a pseudohypoxia state^32^. The reduced growth, observed in mutants, may be indeed included among the Hif-Hypoxia signalling-induced adaptations to energy depletion^33^. These aspects are of scientific and medical relevance, as these signalling cascades and their transducers, shown here to be hyperactivated at embryonic stages, may represent early diagnostic signs and candidate processes for molecularly targeted therapies in POLG disorders.

Our set of multiple POLG models has been also exploited to analyse a series of multiorgan defects that characterize the complex spectrum of POLG disorders. Most notably, the transient downregulation of Polg in zebrafish can already induce at embryonic stages a peculiar enlargement of the cardiac atrium, associated with increased heart rate. Taking advantage of adult-viable *polg*^*sa9574*^ mutants, dilated heart and reduction of the trabecular network could be observed and confirmed at advanced developmental stages; interestingly, dilated cardiomyopathy has been included among the POLG-associated signs^34^. Moreover, as liver and skeletal muscle represent two of the most affected tissues in POLG patients, we focused our attention on these anatomical districts, detecting nonisometric reduction of the hepatic tissue, and skeletal muscle defects including altered birefringence properties of the muscle fibres, decrease of the mitochondrial mass and aberrant mitochondrial cristae. The histopathological phenotypes at the cardiac, hepatic and skeletal muscle level occur in parallel with a net decrease of *polg* mRNA levels, as shown by our expressional analysis in these tissues, suggesting potential mRNA decay or retrograde signalling mechanisms involving the *polg* locus. As the severity of the mtDNA depletion is known to be tissue-specific and in correlation with respiration^6^, this could explain our histopathological findings, supporting altogether the zebrafish organism as a suitable system to faithful model multiorgan defects in POLG-related diseases.

In addition, considering that *POLG* mutations may underlie male infertility and premature menopause in human patients, and that *C. elegans* POLG models show sterility and gonadal dysgenesis^8^, we included in our investigation the reproductive system of zebrafish *polg* mutants. The histological analysis of both female and male gonads highlighted a set of defects, ranging from altered maturation of ovarian follicles to testicular hypoplasia and reduced sperm viability. Interestingly, this suggests an extremely conserved role for Polg activity in gonadal development and function, from both invertebrate and non-mammalian vertebrate models, up to human patients.

Concerning the mitochondrial functionality in zebrafish *polg* mutants, this was ascertained through both indirect and direct assays, measuring mitochondrial mass, morphology and mtDNA content, as well as superoxide production, ROS signalling activation, oxygen consumption and Complex I activity. All these parameters, without any exception, converged towards the identification of a significant decrease of mitochondrial function, the leading pathogenetic mechanism in POLG-related disorders. Indeed, and not surprisingly, mutations in a fundamental gene involved in mtDNA maintenance, such as *POLG*, are expected to lead to a progressive depletion of mtDNA and thus of mtDNA-encoded proteins for oxidative phosphorylation (OXPHOS) and electron transport chain, causing Complex I deficiencies in POLG patients as well as in model systems^35^.

Being these dysfunctions the molecular aspects most desirably targetable by a therapeutic approach, we took advantage of preliminary evidences for an effective activity of the drug CLO in rescuing mtDNA depletion in yeast-, worm- and human fibroblast-based models for POLG disease^8^. In our study, after systemic treatment of mutant zebrafish with CLO, we could define a whole-body non-toxic dosage able to successfully rescue mtDNA quantity to normal levels, even when the mutant phenotype was strongly exacerbated by Ethidium Bromide treatment. Interestingly, this CLO activity proved to be so effective as to induce significant effects also in normal individuals, where the amount of mtDNA could undergo a 25% increase of the basal level. Moreover, the efficacy of CLO was further demonstrated by its ability to rescue Complex I activity in zebrafish *polg* mutants; this effect was so pronounced as to induce at least a 33% increase in both normal and mutated individuals. In parallel, a drastic decrease of Polg-induced retrograde signalling was observed in mutant larvae after CLO treatment. Finally, the drug proved to be also effective in restoring Polg-related cardio-skeletal defects, assessed in terms of skeletal muscle birefringence and mitochondrial mass, cardiac frequency and atrium size. To our knowledge, these are the first evidences of CLO-induced mitochondrial and cardio-skeletal rescue successfully obtained at the whole vertebrate level; overall, these results appear very encouraging in view of a systemic therapy in human POLG patients.

The CLO molecule is an antiarrhythmic agent belonging to Class III as it acts as a Potassium (K^+^) channel blocker. A still unknown target of CLO, related to mtDNA maintenance, may exist, with high conservation in yeast, invertebrates and vertebrates, since CLO treatment leads to similar effects in different in vivo models. In this regard, the zebrafish organism can represent an excellent system where to test candidate mechanisms for CLO activity, ranging from the stabilization of the Polg enzyme to the control of the intracellular ion homeostasis. Among other known Class III antiarrhythmic agents, ibutilide and dofetilide share common chemical structure with CLO^8^. As these drugs proved to be effective in the treatment of POLG worm models, although not at levels comparable with CLO^8^, it would be interesting to test them in a vertebrate setup. Future directions could also point to the analysis of CLO efficacy in rescuing tissue-specific phenotypes, such as hepatic, locomotor or gonadal dysfunction. For instance, since CLO treatment in worm models has been shown to induce an increase of laid eggs^8^, and considering the crucial role of mitochondria in germ-cell selection and gametic function^36^, it would be tempting to verify if CLO can at least partially rescue ovary dysmorphology and sperm viability in zebrafish mutants. Finally, with these zebrafish *polg* lines, now validated and stably maintained, more long-term experiments could be setup, including regeneration assays and multigeneration follow-ups, to investigate the aging process under POLG dysfunction.

In conclusion, we believe that this set of zebrafish-based *POLG* mutants, with either strong or mild phenotypes, may represent precious tools in an experimental pipeline that, taking advantage of unicellular (yeast), invertebrate (worm) and vertebrate (zebrafish) models, can systematically exploit these systems at the best of their potential for comparative phenotyping and large-scale screens for new POLG-targeted therapies.

## Supplementary information

Supplementary Figure Legends

Supplementary Figure 1

Supplementary Figure 2

Supplementary Figure 3

Supplementary Figure 4

Supplementary Figure 5

Supplementary Table 1

## Data Availability

Fish lines used in this study are available through an MTA.
