# Supplementary material for: Efficient clofilium tosylate-mediated rescue of POLG-related disease phenotypes in zebrafish
Source: Cell Death Dis. 2021 Jan 19;12(1):100. doi: 10.1038/s41419-020-03359-z (PMC7815880; doi:10.1038/s41419-020-03359-z)
Supplement: Supplementary file 1 — Supplementary Figure Legends [file 41419_2020_3359_MOESM1_ESM.docx]

Supplementary Figure Legends

**Supplementary Figure 1: *polg* gene knock-down increases CREB signalling**

(A,B,C) Morpholino-mediated knock-down of *polg* (MO) increases CREB signalling (B), compared to mismatched MO-injected (mMO) controls (A), particularly in skeletal muscle and heart region (asterisks), as shown by the CREB-responsive *CRE:mCherry* transgene. (C) Chart reporting the CREB reporter fluorescence relative intensity (R.I.) for the considered conditions. Statistical test: one-way ANOVA followed by Tukey’s test; ***=p<0.0001; N=6 measurements per condition.

**Supplementary Figure 2: *polg* mutants genotyping and *polg* expression analysis**

(A) Representative gel image of PCR genotyping using genomic DNA from tail fins of adults born from a cross between *polg^+/ia302^* heterozygotes. (B) Chromatograms corresponding to *polg^+/+^* and *polg^ia302/ia302^* individuals; the sequence deleted in the mutant is highlighted. (C) High-resolution melt (HRM) analysis allows distinction between genotypes of the *polg^sa9574^* line. HRM curves are shown for each genotype: *polg^+/+^* (blue curve), *polg^+/sa9574^* (green curve) and *polg^sa9574/sa9574^* (red curve). (D) Chromatograms corresponding to *polg^+/+^*, *polg^+/sa9574^* and *polg^sa9574/sa9574^* individuals. The arrow indicates the target site, not mutated in the wt, with two superposed peaks in the heterozygote, and with the substituted base in the mutant. (E) *polg* expression is decreased in all considered tissues of adult *polg^sa9574/sa9574^* mutants, compared to wt individuals. *=p<0.05; N=2; all samples are normalized to pancreatic *polg* levels in wt (reference value set to 1).

**Supplementary Figure 3: Reduction of superoxide levels and ROS signalling in *polg* mutants**

(A,B,C) Reduction of superoxide levels in the head of 2 dpf homozygous *polg^sa9574/sa9574^* mutants (*polg^-/-^*) compared to age-matched controls (*polg^+/+^*), as shown by whole-mount live staining with the red fluorescent indicator MitoSOX; signal quantification is shown in C. Error bars represent the mean ± SEM, *=*p*<0.05; N=5 animals per genotype. (D) Reduction of ROS-induced genes *nqo1* and *txnrd3* in *polg^sa9574/sa9574^* mutants compared to *polg^+/+^* controls. R.I.: Relative Intensity; *=p<0.05; ***=p<0.001; N=6 measurements per condition.

**Supplementary Figure 4: *polg^ia302^* mutants display alterations in myofibrils, locomotion, ROS and retrograde signalling**

(A) Reduced muscle birefringence in 3 dpf *polg^ia302/ia302^* mutants compared to heterozygous and wt siblings. (A’) Relative intensity (R.I.) quantifications of experiments shown in A; N=10 per genotype. (B,C) Average distance (in mm) for each 2-min interval swum by larvae under light-dark period (dark period in grey) at 6 dpf (B) and 15 dpf (C). Heterozygous and homozygous *polg^ia302^* mutants display reduced locomotion at 15 dpf, compared to wt siblings. (B’,C’) Charts on experiments shown in B and C, with total distance (in mm) swum by larvae for 1 hour at 6 dpf (B’) and 15 dpf (C’); N=23 for 6 dpf; N=36 for 15 dpf. (D) Analysis of mtDNA levels (*nd1* relative abundance) in heart, brain, gonads and skeletal muscle, fails to detect significant differences between *polg^+/+^* and *polg^+/ia302^* individuals. (E,F) Retrograde and ROS signalling analysis in heart (E) and brain (F) of adult *polg^+/+^* and *polg^+/ia302^* individuals detects in heterozygotes increased Hif-Hypoxia (*pfkfb3*, *hbbe3*) and CREB (*fosab*) signalling activation, in parallel with a decrease of ROS-induced *nqo1* expression; N=5. All charts in Suppl. Fig. 4 display data as mean ± SEM; n.s.=not significant; *=p<0.05; **=p<0.01.

**Supplementary Figure 5*:* CLO toxicity analysis in zebrafish**

(A) Kaplan-Meier survival curves of wt larvae in the presence of CLO at 2.5, 5.0, 10.0, 20.0 µM and with no treatment. No wt fish exposed to CLO at 20 µM could survive up to 3 days of treatment. (B,C,D,E) Overt dysmorphology is observed at CLO dosages over 5.0 µM.

**Supplementary Table 1: List of oligomers used in this study**
