# Supplementary figures and images for: Efficient clofilium tosylate-mediated rescue of POLG-related disease phenotypes in zebrafish
Source: Cell Death Dis. 2021 Jan 19;12(1):100. doi: 10.1038/s41419-020-03359-z (PMC7815880; doi:10.1038/s41419-020-03359-z)

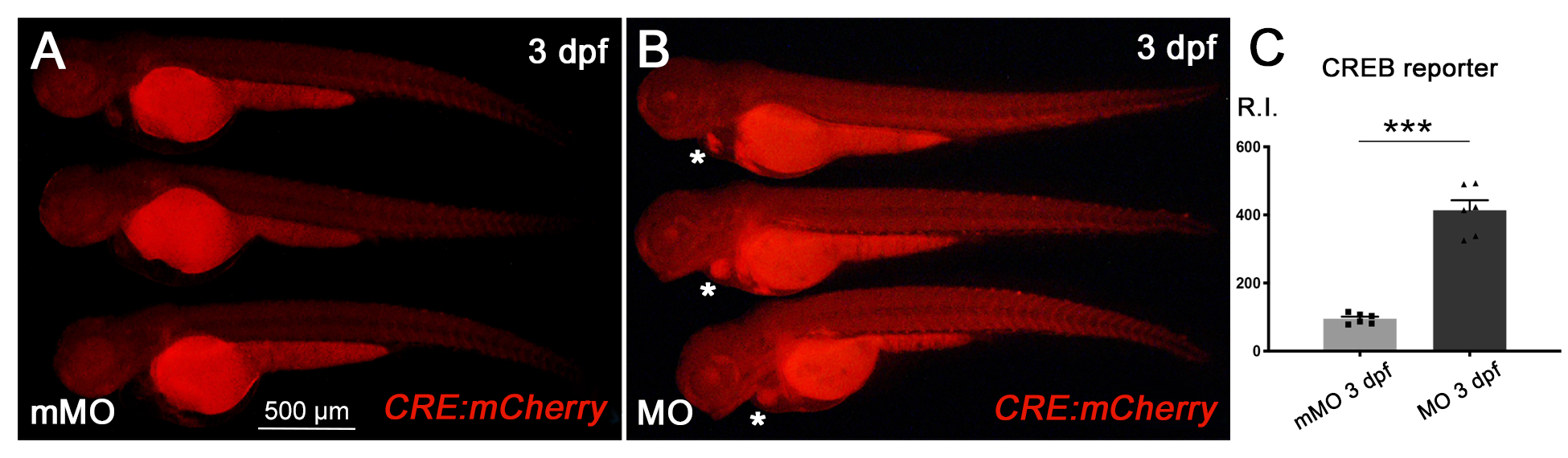

Supplement: Supplementary file 2 — Supplementary Figure 1 [file 41419_2020_3359_MOESM2_ESM.tif]

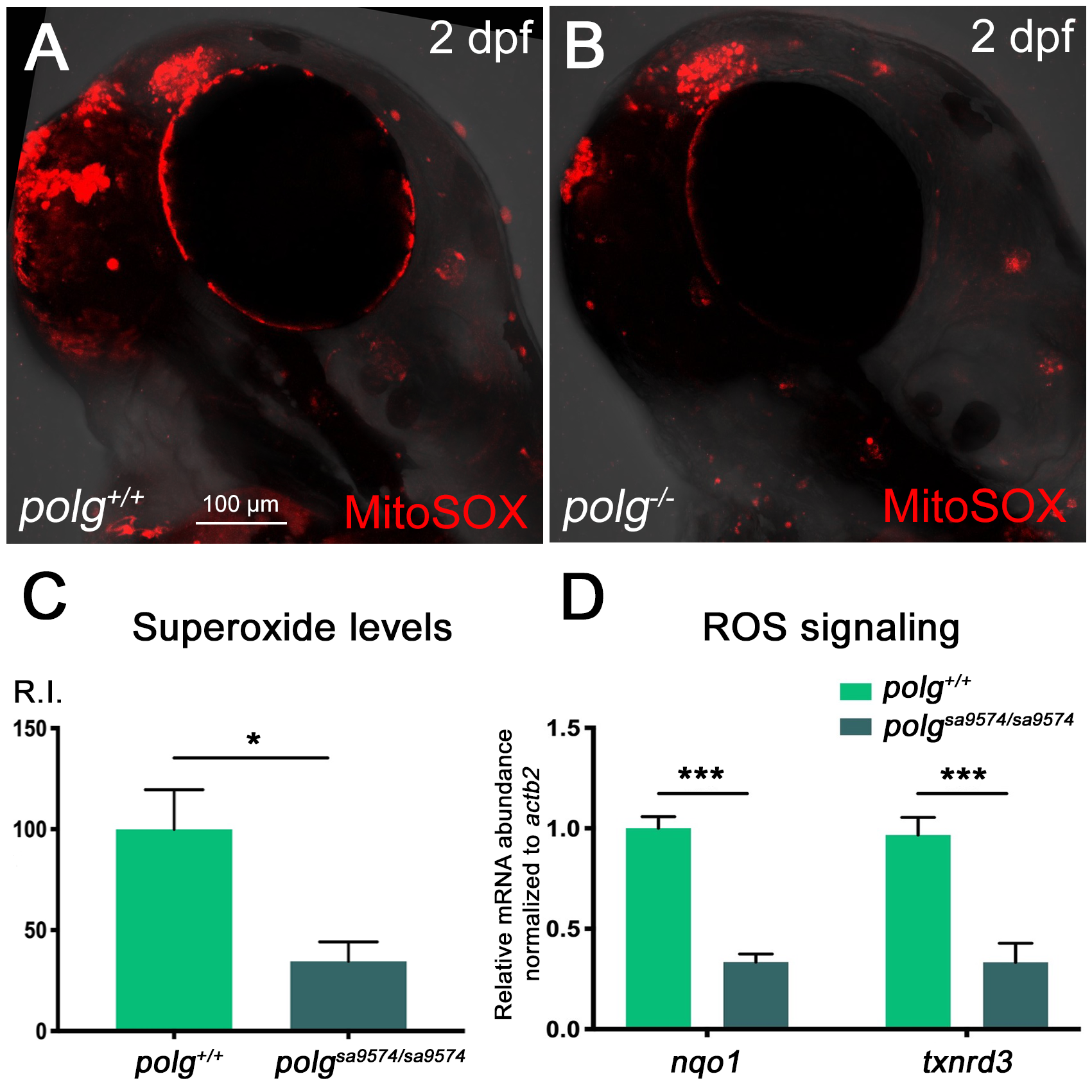

Supplement: Supplementary file 4 — Supplementary Figure 3 [file 41419_2020_3359_MOESM4_ESM.tif]

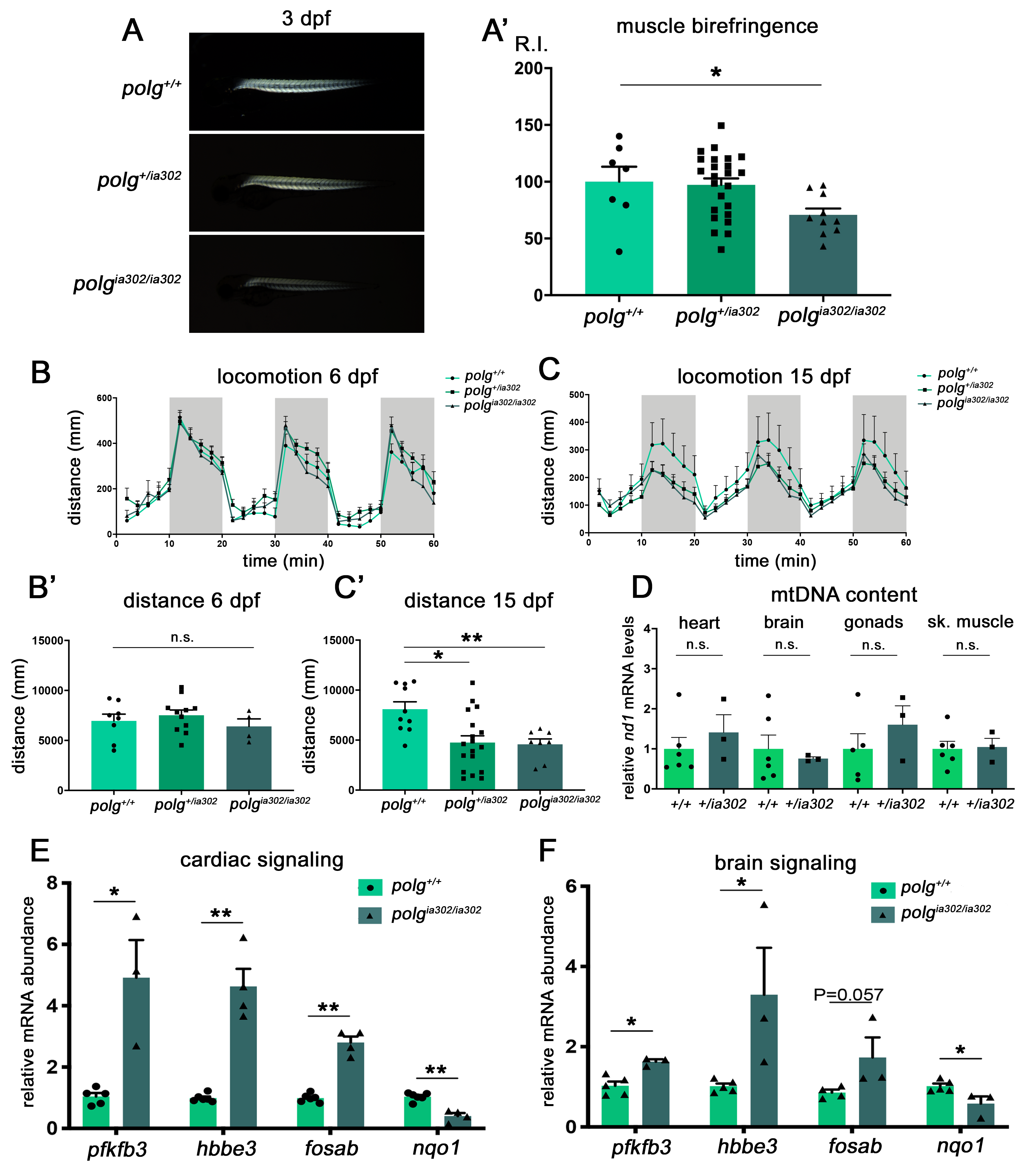

Supplement: Supplementary file 5 — Supplementary Figure 4 [file 41419_2020_3359_MOESM5_ESM.tif]

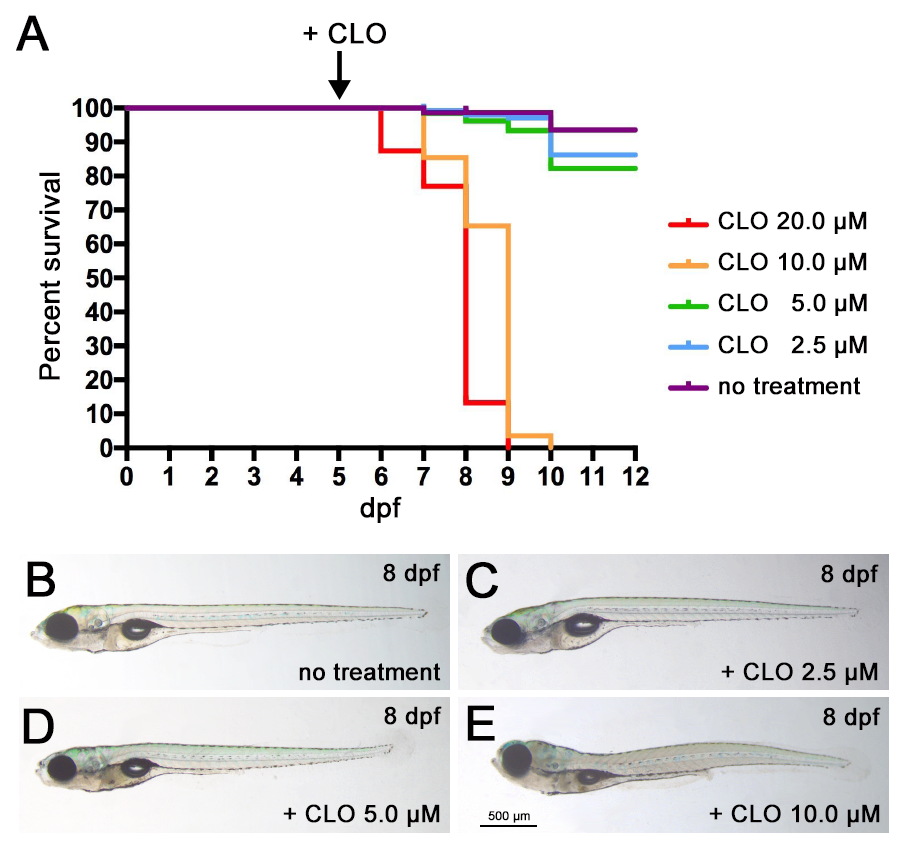

Supplement: Supplementary file 6 — Supplementary Figure 5 [file 41419_2020_3359_MOESM6_ESM.tif]
