# Supplementary material for: Efficient clofilium tosylate-mediated rescue of POLG-related disease phenotypes in zebrafish
Source: Cell Death Dis. 2021 Jan 19;12(1):100. doi: 10.1038/s41419-020-03359-z (PMC7815880; doi:10.1038/s41419-020-03359-z)
Supplement: Supplementary file 7 — Supplementary Table 1 [file 41419_2020_3359_MOESM7_ESM.docx]

| **Oligomer name** | **Gene / Accession No.** | **Type** | **Sequence (5’ - 3’)** | **Effect; Product** |
| --- | --- | --- | --- | --- |
| MO-*polg*-e4i4 | *polg* / ZDB-GENE-060303-1 | splicing morpholino oligomer | AGCTGTGAGCTGACAAACCTTAGGT | *polg* KD, intron keeping |
| mismMO-e4i4 | *polg* / ZDB-GENE-060303-1 | mismatched morpholino oligomer | AGgTgTGAGaTGACAAACaTTAaGT | No effect; negative control |
| MO-*polg*-ATG | *polg* / ZDB-GENE-060303-1 | ATG morpholino oligomer | GACGTAGCATCGTATTAAAGCATGA | *polg* KD |
| MO-*polg*-ex3-F | *polg* / ZDB-GENE-060303-1 | diagnostic DNA primer for MO KD | ATCTCATCCCGCTGGAAAC | Normal product: 510 bp  Intron keeping: ca. 1300 bp |
| MO-*polg*-ex6-R | *polg* / ZDB-GENE-060303-1 | diagnostic DNA primer for MO KD | GGGGAAGCTGTTGAGTGAAG |  |
| *actb1-F* | *actb1* / ZDB-GENE-000329-1 | control DNA primer for MO KD | TGTTTTCCCCTCCATTGTTGG | From cDNA: 558 bp  From genomic DNA: 1268 bp |
| *actb1-R* | *actb1* / ZDB-GENE-000329-1 | control DNA primer for MO KD | TTCTCCTTGATGTCACGGAC |  |
| Constant oligo | *polg* / ZDB-GENE-060303-1 | Crispr/Cas9 DNA oligomer for gRNA | AAAAGCACCGACTCGGTGCCACTTTTTCAAGTTGATAACGGACTAGCCTTATTTTAACTTGCTATTTCTAGCTCTAAAAC | Crispr/Cas9-induced  *polg* mutagenesis |
| *polg*-specific oligo | *polg* / ZDB-GENE-060303-1 | Crispr/Cas9 DNA oligomer for gRNA  (**gRNA sequence**) | ATTTAGGTGACACTATA**GGGGCGGTGAGTGGAAGGAG**GTTTTAGAGCTAGAAATAGCAAG | Crispr/Cas9-induced  *polg* mutagenesis |
| *polg*-*sa9574*-F1 | *polg* / ZDB-GENE-060303-1 | DNA primer for *sa9574* genotyping | TAGAGATCACCAAGGGCTCG | From F1-R1 pair: 82 bp  From F2-R1 pair: 108 bp |
| *polg*-*sa9574*-F2 | *polg* / ZDB-GENE-060303-1 | DNA primer for *sa9574* genotyping | CGAGGCGCTGGACATTTATC |  |
| *polg*-*sa9574*-R1 | *polg* / ZDB-GENE-060303-1 | DNA primer for *sa9574* genotyping | GAGAGCCGCTCATCCCACAG |  |
| *polg*-*ia302*-F1 | *polg* / ZDB-GENE-060303-1 | DNA primer for *ia302* genotyping | ATCGGTATGCCTGGTCAAGT | Normal product: 151 bp  Deleted product: 135 bp |
| *polg*-*ia302*-R1 | *polg* / ZDB-GENE-060303-1 | DNA primer for *ia302* genotyping | TGCTCTTTAATGTGTGCCCG |  |
| *rplp0-F* | *rplp0 / ZDB-GENE-000629-1* | DNA primer for Real Time RT-PCR | CTGAACATCTCGCCCTTCTC | Housekeeping gene  cDNA control product: 161 bp |
| *rplp0-R* | *rplp0 / ZDB-GENE-000629-1* | DNA primer for Real Time RT-PCR | TAGCCGATCTGCAGACACAC |  |
| *actb2-RT-F* | *actb2* / ZDB-GENE-000329-3 | DNA primer for Real Time RT-PCR | TGGGTATGGAATCTTGCGGT | Housekeeping gene  cDNA control product: 198 bp |
| *actb2-RT-R* | *actb2* / ZDB-GENE-000329-3 | DNA primer for Real Time RT-PCR | GTGGGGCAATGATCTTGATCT |  |
| *hbbe3*-F | *hbbe3 /* ZDB-GENE-980526-287 | DNA primer for Real Time RT-PCR | CTCAGCGAGCTTCACTCAGA | Hif-Hypoxia signaling target cDNA target product: 143 bp |
| *hbbe3*-R | *hbbe3 /* ZDB-GENE-980526-287 | DNA primer for Real Time RT-PCR | GACAGGAACTTCTGCCAAGC |  |
| *pfkfb3*-F | *pfkfb3 /* ZDB-GENE-040426-2724 | DNA primer for Real Time RT-PCR | GCAAACCCTCCAACAGTGAT | Hif-Hypoxia signaling target cDNA target product: 148 bp |
| *pfkfb3*-R | *pfkfb3 /* ZDB-GENE-040426-2724 | DNA primer for Real Time RT-PCR | GTTTCACTGCTTCACGACGA |  |
| *fosab*-F | *fosab /* ZDB-GENE-031222-4 | DNA primer for Real Time RT-PCR | CGCTCAACCAGACTCAGGAG | CREB signaling target  cDNA target product: 80 bp |
| *fosab*-R | *fosab /* ZDB-GENE-031222-4 | DNA primer for Real Time RT-PCR | TGAAGAGATCGCCGTGACAG |  |
| *nqo1-F* | *nqo1 /* ZDB-GENE-030131-1226 | DNA primer for Real Time RT-PCR | AGAGGGCCATGCTTTCCTTC | ROS-induced gene  cDNA target product: 114 bp |
| *nqo1-R* | *nqo1 /* ZDB-GENE-030131-1226 | DNA primer for Real Time RT-PCR | AACGCAGCACTCCATTCTGT |  |
| *txnrd3-F* | *txnrd3 /* ZDB-GENE-030327-3 | DNA primer for Real Time RT-PCR | CCCACCACTGTCTTCACTCC | ROS-induced gene  cDNA target product: 127 bp |
| *txnrd3-R* | *txnrd3 /* ZDB-GENE-030327-3 | DNA primer for Real Time RT-PCR | CGGTAAACTCCAGAGGCCAG |  |
| *nucl-polg*-F | *polg* / ZDB-GENE-060303-1 | primer for mtDNA depletion analysis | GAGAGCGTCTATAAGGAGTAC | Reference nuclear gene  Genomic DNA product: 81 bp |
| *nucl-polg*-R | *polg* / ZDB-GENE-060303-1 | primer for mtDNA depletion analysis | GAGCTCATCAGAAACAGGACT |  |
| *mt-nd1*-F | *mt-nd1* / ZDB-GENE-011205-7 | primer for mtDNA depletion analysis | AGCCTACGCCGTACCAGTATT | Reference mitochondrial gene Mt DNA product: 143 bp |
| *mt-nd1*-R | *mt-nd1* / ZDB-GENE-011205-7 | primer for mtDNA depletion analysis | GTTTCACGCCATCAGCTACTG |  |
